# The First High-quality Reference Genome of Sika Deer Provides Insights into High-tannin Adaptation

**DOI:** 10.1016/j.gpb.2022.05.008

**Published:** 2022-06-16

**Authors:** Xiumei Xing, Cheng Ai, Tianjiao Wang, Yang Li, Huitao Liu, Pengfei Hu, Guiwu Wang, Huamiao Liu, Hongliang Wang, Ranran Zhang, Junjun Zheng, Xiaobo Wang, Lei Wang, Yuxiao Chang, Qian Qian, Jinghua Yu, Lixin Tang, Shigang Wu, Xiujuan Shao, Alun Li, Peng Cui, Wei Zhan, Sheng Zhao, Zhichao Wu, Xiqun Shao, Yimeng Dong, Min Rong, Yihong Tan, Xuezhe Cui, Shuzhuo Chang, Xingchao Song, Tongao Yang, Limin Sun, Yan Ju, Pei Zhao, Huanhuan Fan, Ying Liu, Xinhui Wang, Wanyun Yang, Min Yang, Tao Wei, Shanshan Song, Jiaping Xu, Zhigang Yue, Qiqi Liang, Chunyi Li, Jue Ruan, Fuhe Yang

**Affiliations:** 1 Key Laboratory of Genetics, Breeding and Reproduction of Special Economic Animals, Ministry of Agriculture and Rural Affairs, Institute of Special Animal and Plant Sciences, Chinese Academy of Agricultural Sciences, Changchun 130112, China; 2 Shenzhen Branch, Guangdong Laboratory of Lingnan Modern Agriculture, Genome Analysis Laboratory of the Ministry of Agriculture and Rural Affairs, Agricultural Genomics Institute at Shenzhen, Chinese Academy of Agricultural Sciences, Shenzhen 518120, China; 3 CAS Key Laboratory of Forest Ecology and Management, Institute of Applied Ecology, Chinese Academy of Sciences, Shenyang 110016, China; 4 Annoroad Gene Technology (Beijing) Co., Ltd, Beijing 100176, China; 5 Novogene Bioinformatics Institute, Beijing 100083, China

**Keywords:** Sika deer, Whole-genome sequencing, Chromosome-scale assembly, Oak leaf, Tannin tolerance

## Abstract

**Sika deer** are known to prefer **oak leaves**, which are rich in tannins and toxic to most mammals; however, the genetic mechanisms underlying their unique ability to adapt to living in the jungle are still unclear. In identifying the mechanism responsible for the tolerance of a highly toxic diet, we have made a major advancement by explaining the genome of sika deer. We generated the first high-quality, chromosome-level genome assembly of sika deer and measured the correlation between tannin intake and RNA expression in 15 tissues through 180 experiments. Comparative genome analyses showed that the *UGT* and *CYP* gene families are functionally involved in the adaptation of sika deer to high-tannin food, especially the expansion of the *UGT* family 2 subfamily B of *UGT* genes. The first chromosome-level assembly and genetic characterization of the tolerance to a highly toxic diet suggest that the sika deer genome may serve as an essential resource for understanding evolutionary events and tannin adaptation. Our study provides a paradigm of comparative expressive genomics that can be applied to the study of unique biological features in non-model animals.

## Introduction

**Figure 1:**
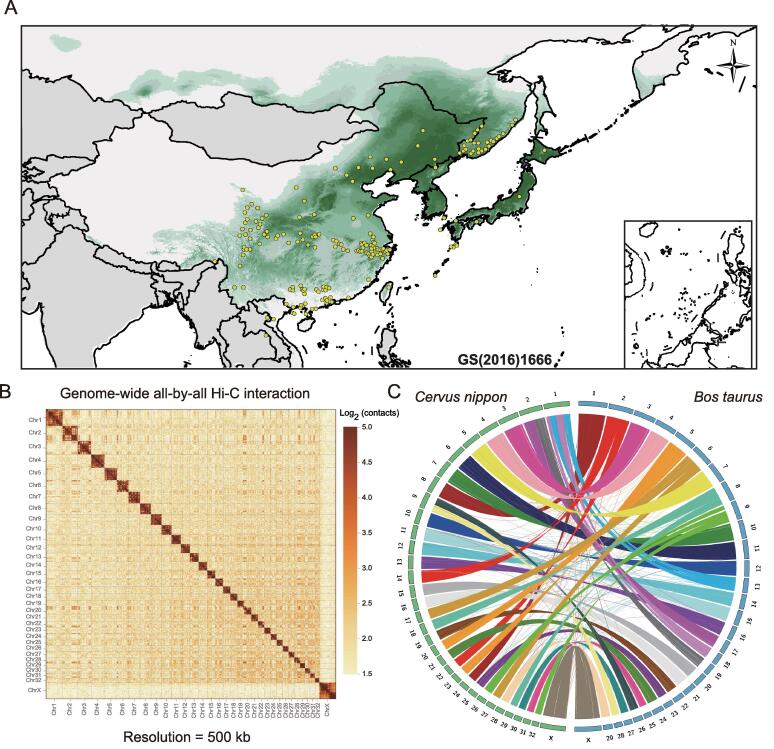
**Distribution and genome assembly of sika deer** **A.** Mongolian oak and sika deer distribution. The green shadow represents the distribution range of Mongolian oak. The yellow dots represent the historical distribution of sika deer in 5 countries (China, Russia, Japan, North Korea, and Vietnam). **B.** A contact map at a 500-kb resolution of chromosome-level assembly in sika deer is shown. The color bar illuminates the logarithm of the contact density from red (high) to white (low) in the plot. Note that only sequences anchored on chromosomes are shown in the plot. **C.** Synteny analysis of cattle and sika deer. Circular graphs displaying the results of the synteny analysis. Same-color ribbons connect syntenic genomic segments. Hi-C, high-throughput chromosome conformation capture.

Cervidae consists of 55 extant deer species and constitutes the second largest family in terrestrial artiodactyls. Sika deer (*Cervus nippon*) is naturally distributed throughout East Asia and is one of the best-known deer species producing velvet antlers [1], [2], a valuable ingredient in traditional Chinese medicine [3]. Among other deer species [4], [5], [6], sika deer has unique characteristics, such as a geographic distribution that is significantly more coincident with oak trees (Figure 1A) and an ability to tolerate a high-tannin diet, mainly consisting of oak leaves. Notably, oak leaves, which are rich in tannins and toxic to most mammals, such as cattle [7], are conversely found to increase the reproductive rate and fawn survival rate of sika deer in the breeding process of some farmers. Thus, oak leaves are essential for maintaining healthy sika deer in wild and farmed populations. Some studies have claimed that tannins are not toxic to sika deer because of the rumen microbes and fermentation patterns of these deer [8]. However, knowledge is scarce regarding the genetics and mechanism underlying the ability to detoxify a high-tannin diet.

Whole-genome sequencing has become a popular technology which can be used to explore the taxonomy, evolution, and biological phenomena of organisms at the molecular level [9], compared with morphological, histological, and other analyses [10], [11], [12]. For example, a series of studies have investigated genomes of 11 deer and 33 other ruminant species and identified some genes that are involved in some special biological processes, such as ruminant headgear formation, rapid antler regeneration, and reindeer adaptation to the long days and nights in the Arctic region [6], [13], [14]. The chromosome-level genome for sika deer is in high demand compared with that for other ruminants such as bovines [15], [16]. It will provide novel insights and molecular evolutionary information on the exceptional characteristics of the sika deer.

Here, we report the chromosome-level genome assembly of a female sika deer, as well as the RNA sequencing of 15 tissue types in sika deer treated with 3 levels of a high-tannin diet. The findings provide important resources to help elucidate the genetic mechanisms underlying the high-tannin food tolerance of sika deer. Our high-quality sika deer genome will be of great importance to researchers who study the common characteristics of deer and other ruminants and could even serve as a reference deer genome. The well-designed RNA expression experiments used in this study also provide a paradigm for studying novel features in nonmodel animals.

## Results

### *De novo* assembly of a *C. nippon* reference genome

We collected DNA from a female sika deer and identified a total of 66 chromosomes, including 64 autosomes and one pair of sex chromosomes (XX) (Figure S1). A large set of data was acquired for assembly using a combination of four technologies. (1) A total of 242.9 Gb of clean data (∼ 93.4×) were obtained from paired-end sequencing (Illumina HiSeq), with the genome size (2.6 Gb) estimated by the 25-mer distribution (Figure S2; Table S1). (2) A total of 150.4 Gb (∼ 57.7×) of PacBio RSII long reads [single-molecule real-time (SMRT) sequencing] were also acquired (Table S2). The wtdbg (v1.2.8) [17] assembler yielded 2040 primary contigs using PacBio reads with a contig N50 size of 23.6 Mb and the longest at 93.6 Mb (Table S3). These contigs were then polished. A genome-wide base-level correction was performed and the inconsistencies between the polished genome and the Illumina short reads were identified and corrected by in-house script to produce a highly accurate assembly. (3) The previous contigs were clustered into chromosome-scale scaffolds using high-throughput chromosome conformation capture (Hi-C) proximity-guided assembly (Figure 1B) to produce the final reference assembly, named MHL_v1.0, totaling 2.5 Gb of sequence with a contig N50 of 23.6 Mb and a scaffold N50 of 78.8 Mb (Table 1). The resulting assembly contained 2,481,763,803 bp reliably anchored on chromosomes, accounting for 99.24% of the whole genome (Table S4). (4) A total of 264 Gb of optical mapping (using BioNano Genomics Irys) data were also used to generate *de novo*-assembled optical maps with a scaffold N50 of 1.974 Mb, which was sequentially compared with MHL_v1.0 to identify the misoriented contigs and improve the final validated reference assembly (Figure S3).

**Table 1:** **Comparison of genome quality and annotation between the genome of sika deer and the best published genome of red deer**

		**Sika deer** **(*Cervus nippon*)**	**Red deer** **(*Cervus elaphus*)**
Assembly	Total sequence length (bp)	2,500,646,934	3,438,623,608
	Total length without gaps (bp)	2,500,501,634	1,960,832,178
	Number of scaffolds	588	11,479
	Scaffold N50 (bp)/L50	78,786,809/12	107,358,006/13
	Number of contigs	2040	406,637
	Contig N50 (bp)/L50	23,559,432/33	7944/64,532
	Total number of chromosomes	33	35
	Anchored rate	99.24%	98.33%
Annotation	Number of genes	21,449	19,243
	Average gene length (bp)	39,397.69	28,008.84
	Average CDS length (bp)	1617.26	1085.04
	Average number of exons per gene	9.29	6.5
	Average exon length (bp)	174.03	167.06
	Average intron length (bp)	4555.82	4755.75

To validate our assembly, MHL_v1.0 was compared with the previously published red deer (*Cervus elaphus*) [18] genome (Figure S4). Both the inconsistency of the synteny analysis and the improper density of Hi-C proximity maps identified 34 inaccurate junctions, which were considered potential inversions and misassemblies (Figures S4 and S5). The aforementioned optical maps were used to determine whether the 34 inaccurate junctions were breakpoints or new joint regions after the replacement. We found that 10 inaccurate junctions were supported by the optical maps, and those junctions were then manually inspected and correlated. Additionally, another 142 potential misjoined contigs were found by comparing our MHL_v1.0 assembly with the optical maps. The paired-end Illumina short reads were then mapped to the final assembly, and all 142 disagreements were checked manually and found to be sequential in the comparison results. We further compared MHL_v1.0 with the twenty published genomes of Cervidae, including red deer and reindeer (*Rangifer tarandus*). The results showed that the MHL_v1.0 chromosome-level assembly was more accurate than those previously published (Table S5). We detected the fission/separation events of MHL_v1.0 and compared three chromosome-level ruminant genomes [UMD_3.1 (cattle), ARS1 (goat), and CerEla1.0 (red deer) downloaded from National Center for Biotechnology Information (NCBI)] with sika deer. The results showed that the sika deer genome had the highest chromosome collinearity with red deer (Figure 1C, Figure S6).

Finally, we downloaded a total of 2715 expressed sequence tag (EST) sequences belonging to sika deer from the NCBI dbEST database and aligned them against MHL_v1.0. We found that 95.95% of the EST sequences (coverage rate > 90%) matched our sika deer genome MHL_v1.0. Evaluation of our MHL_v1.0 using CEGMA (v2.5) [19] software showed that 97.18% of the full length of 248 genes in the core gene set was predicted. Benchmarking Universal Single-Copy Orthologs (BUSCO) (v3.1.0, OrthoDB v9) analysis of the gene set showed that complete BUSCO accounted for 3880 (of 4,104; 94.60%) genes, which is better than the results obtained for the water buffalo (*Bubalus bubalis*, 93.6%) [12] and domestic goat (*Capra hircus*, 82%) [20]. After aligning Illumina short reads (∼ 93.4×) against MHL_v1.0, the base level error rate was estimated to be 1.1E–5 (Table S6).

### Composition of transposable elements and genome annotation

Homology and *de novo* repetitive sequence annotation results showed that repetitive sequences accounted for approximately 45.38% of MHL_v1.0, which is consistent with the percentages published for other mammals (Tables S7 and S8), including humans (44.83%) [21], water buffalo (45.33%) [12], and sheep (42.67%) [22]. As in other published mammalian genomes, long interspersed nuclear elements (LINEs), short interspersed nuclear elements (SINEs), and long terminal repeats (LTRs) were also the most abundant elements in MHL_v1.0 (29.56%, 7.63%, and 5.38% of the total number of elements, respectively) (Figure S7). The main features of MHL_v1.0 are summarized and shown in Figure S8.

A total of 21,449 protein-coding genes were predicted using the combined methods of homology and *de novo* annotations with transcriptome data (mapping rate of 93.43% for 1.2 billion RNA-Seq reads), and 90.1% of the protein-coding genes were functionally annotated (Table S9). The average coding sequence (CDS) length per gene was 1617 bp, the exon number per gene was 9.29, and the average length per exon was 174 bp; these values are similar to those in other mammals (Table S10). To verify the accuracy of our gene predictions and to assess the annotation completeness of MHL_v1.0, we checked core gene statistics using the BUSCO software. A total of 3907 (of 4104; 95.20%) (Table S11) highly conserved core proteins in mammals were recovered from our predictions.

### Divergence time and population changes in different periods of sika deer

**Figure 2:**
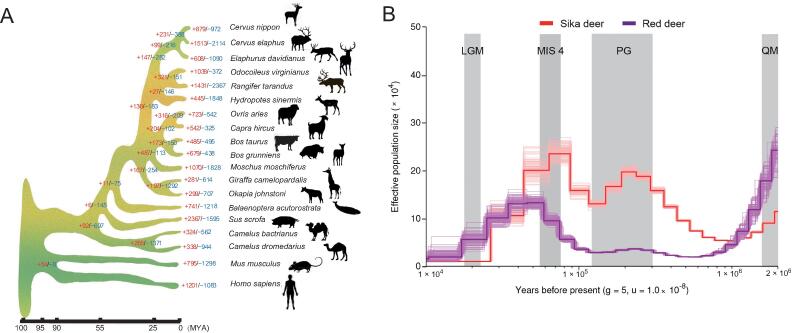
**Evolutionary analysis of sika deer** **A.** Phylogenetic tree inferred from 19 species. The X-axis is the inferred divergence time based on the phylogenetic tree and fossils. The number of expanded gene families is red, and the number of contracted gene families is blue. **B.** PSMC analysis of effective population sizes in sika deer and red deer. The generation times (g) is 5 years and the per generation mutation rate (u) is 1.0E–8. LGM, last glacial maximum (∼ 0.02 MYA); MIS 4, marine oxygen isotope stage 4 (0.05–0.075 MYA); PG, penultimate glaciation (0.13–0.3 MYA); QM, Qingzang movement (1.7–3.6 MYA); PSMC, pairwise sequential markovian coalescent; MYA, million years ago.

A phylogenetic tree (Figure 2A) based on 19 mammals spanning the orders Primates, Rodentia, Artiodactyla, and Cetacea was constructed with the maximum likelihood (ML) method using 748 identified single-copy orthologous genes. The results showed that sika deer was in the same clade as red deer (Figure 2A), which is consistent with the cladistic data [23]. The divergence time between sika deer and red deer was estimated to be approximately 2.5 million years ago (MYA) (Figure 2A, Figure S9).

To examine the changes in effective population size (*N*_e_) of the ancestral populations, a pairwise sequential Markovian coalescent (PSMC) [24] analysis was applied to sika deer and red deer [18] (Figure 2B). Demographic analysis showed that the *Ne* of the sika deer sharply declined during the two large glaciations: the Qingzang movement (QM, 1.7–3.6 MYA) and penultimate glaciation (PG, 0.13–0.3 MYA), and the sika deer population dropped to a low level. Subsequently, the *Ne* increased greatly after that period, suggesting that these deer had adapted to the specific habitat, probably due to the monsoon climate in East Asia. During the same period, the population of red deer recovered soon after a decline and shrank again, but the red deer population decreased significantly and then expanded on a small scale. During marine oxygen isotope stage 4 (MIS 4, 0.058–0.074 MYA) and the last glacial maximum (LGM, ∼ 0.02 MYA), the population of sika deer and red deer continuingly decreased again (Figure 2B).

### Positive selection and gene family evolution

We identified a total of 9830 homologous gene families in MHL_v1.0 by comparing the predicted protein sequences of sika deer with those of 19 mammals spanning the orders Primates, Rodentia, Artiodactyla, and Cetacea (Figure S10; Table S12).

Based on the hypothesis that potential genomic adaptations are related to genes that are under positive selection in the sika deer lineages [25], we identified 55 positively selected genes (PSGs), which were calculated using the branch-site models and validated using likelihood ratio tests (Table S13). The PSGs were found to be involved in the PI3K-Akt signaling pathway (ko04151), VEGF signaling pathway (ko04370), and pathways in cancer (ko05200), among others. These pathways were reportedly related to antler growth [26], [27].

The number of genes in a gene family has been proposed as a major factor underlying the adaptive divergence of closely related species. To depict the gene family evolution, we identified 972 contracted and 879 expanded gene families in sika deer compared with other species (Figure 2A; Table S14). *P* value was corrected by Benjamini–Hochberg (BH) method. The expanded gene families were mainly enriched in the signal transduction pathways of environmental perception (olfactory transduction, G protein-coupled receptors, and neuroactive ligand-receptor interaction, BH-adjusted *P* < 0.05), enzymatic activity (transferase activity, transferring hexosyl groups, carboxypeptidase activity, and L-lactate dehydrogenase activity, BH*-*adjusted *P* < 0.05), feeding behavior (salivary secretion and neurotransmitter secretion, BH*-*adjusted *P* < 0.05), and drug metabolism (drug metabolism - other enzymes, drug metabolism - cytochrome P450, and metabolism of xenobiotics by cytochrome P450, BH-adjusted *P* < 0.05) (Tables S15 and S16). The contracted gene families were mainly related to lipid metabolism pathways (linoleic acid metabolism and ether lipid metabolism, BH-adjusted *P* < 0.05), ion transportation (calcium ion binding, anion transport, and iron ion binding, BH-adjusted *P* < 0.05), and regulation of basic biological processes (regulation of developmental and apoptotic processes, BH-adjusted *P* < 0.05) (Tables S17 and S18).

### Exceptional expansion of the uridine 5′-diphospho-glucuronosyltransferase gene family in the sika deer genome

**Figure 3:**
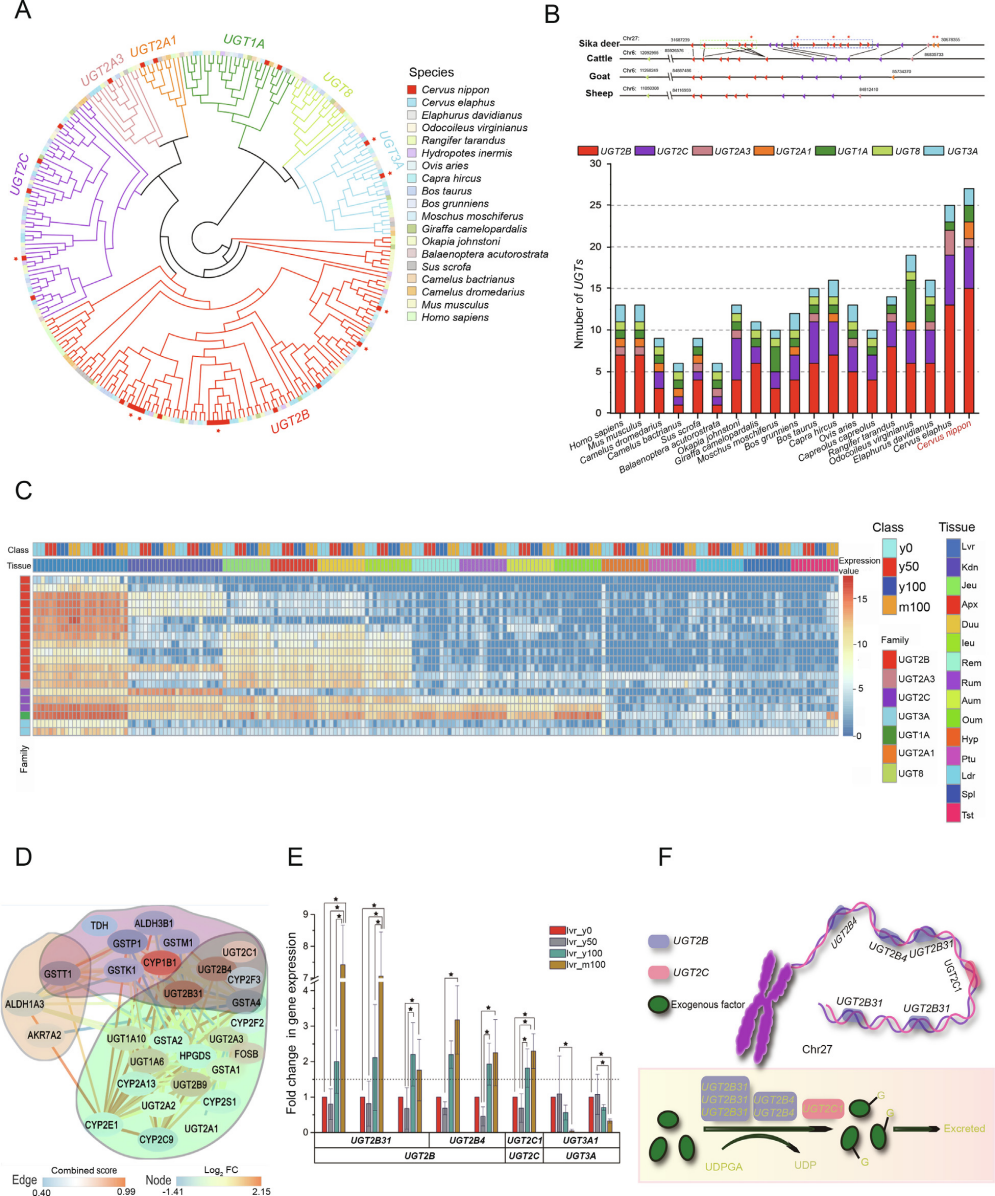
***UGT* expansion and high-tannin adaptation in sika deer** Transcriptome analysis revealed that the *UGT* gene family was the key factor for sika deer adaptation to a high-tannin diet. **A.** Gene tree of *UGTs* in 19 species. Five out of six up-regulation significantly differentially expressed *UGT* genes in sika deer were in *UGT2B* subfamily [red genes are *UGT* genes in sika deer and red stars represent eight differentially expressed *UGT* genes (BH-adjusted *P* < 0.05)]. **B.** Number of *UGT* genes in 19 species and synteny analysis of *UGTs* in sika deer, cattle, goat, and sheep. Synteny analysis shows that 23 of 27 *UGT* genes are distributed in chromesome 27 and all six up-regulated differentially expressed *UGT* genes are distributed in chromesome 27 (red stars). The *UGT* genes are replicated (green dotted box and blue dotted box). And 3 up-regulated differentially expressed *UGT* genes are in the blue box. **C.** Expression heatmap of *UGTs* of sika deer in different tissues and treatments. The abbreviations stand for different tissues: Lvr, liver; Kdn, kydney; Jeu, jejunum; Apx, appendix; Duu, duodenum; Ieu, ileum; Rem, reticulum; Rum, rumen; Aum, abomasum; Oum, omasum; Hyp, hypothalamus; Ptu, pituitary; Ldr, longissimus dorsi; Spl, spleen; Tst, testis. y means young deer samples, m means mature deer samples. **D.** The overlap between 3 contracted genes (yellow background), 20 expanded genes (green background) from contracted or expanded gene families, and 12 DEGs (pink background), which all play a role in the cytochrome P450 pathway. **E.** Expression change of 8 DEGs in sika deer liver resulting from different treatments. A star indicates that the difference in expression between different treatments is significant (BH-adjusted *P* < 0.05). **F.** Schematic of the glucuronidation reaction, showing that six up-regulated *UGT* genes in the *UGT2B* and *UGT2C* subfamilies were located on sika deer chromosome 27. UDPGA, uridine 5′-diphospho-glucuronic acid; DEGs, differentially expressed genes (BH-adjusted *P* < 0.05); *UGT*, 5'-diphospho-glucuronosyltransferase; BH, Benjamini–Hochberg; FC, fold change; Chr, chromosome; G, glucuronosyl; UDP, uridine diphosphate.

Gene gains and losses are one of the primary contributors to functional changes. To better understand the evolutionary dynamics of genes, we assessed the expansion and contraction of the gene ortholog clusters among 19 species. The uridine 5′-diphosphoglucuronosyltransferase (*UGT*) gene families were at the top 27 of 879 significantly expanded gene families, which have been reported to play a role in the detoxification of exogenous compounds [28], [29], [30]. Phylogenetic analysis revealed that the 257 *UGT* genes could be classified into 7 lineages (Figure 3A, Figure S11), while in the sika deer genome, we found two lineage-specific monophyletic expansions of the *UGT* family 2 subfamily B (*UGT2B*) and *UGT* family 2 subfamily C (*UGT2C*). Synteny analysis shows that 23 of 27 *UGT* genes are distributed in chromosome 27 and gene replication can be detected (Figure 3B). In the *UGT2B* subfamily, 15 of all *UGT* genes were found in the sika deer genome, which was more than the number of genes in *UGT2B* subfamily in any other species assessed in this study (Table S19). Sika deer had relatively lower number of expanded genes in the *UGT2C* subfamily than in the *UGT2B* subfamily. The main detoxification reactions are traditionally categorized into phase I and phase II reactions. Currently available evidence indicates that among these, the *CYP*, *UGT*, *GST*, and *SULT* gene families have the greatest importance in xenobiotic metabolism. Taken together, these results prompt us to propose that the exceptional expansion of the *UGT* gene family may be the key genetic basis for the tolerance of high-tannin food, namely, oak leaves, by the sika deer.

### Transcriptome experiments reveal important differentially expressed genes in adaptation of a high-tannin diet

Sika deer adapted well to living in the forest and have consumed a high-tannin diet of Mongolian oak (*Quercus mongolica*) leaves (MOL) for a long time; whether the underlying genetic adaptation and molecular mechanism are associated with the special expansion of *UGT* gene families is an interesting question. We used 9 deer fawns to conduct a feeding trial with different tannin-containing (0%, 50%, and 100%) diets, and 3 mature deer (100%) were used as a comparison group. Transcriptome sequencing was performed on 15 tissues of all experimental individuals (Table S20). A total of 1.44 Tb of transcriptional data from 180 samples were obtained using the Illumina platform, and the 17,233 differentially expressed genes (DEGs) were analyzed by pairwise comparison of each group (Figure S12). The liver is the major organ associated with *UGT* activity, and *UGT* expression was highest in the liver among the fifteen tissues examined (Figure 3C). Although *UGT* genes were also highly expressed in the liver tissue of cattle, they did not respond to high MOL levels (Figure S13). Transcriptome analysis of different class in cattle showed that *UGT* genes were not significantly differentially expressed in the liver (BH-adjusted *P* > 0.05). We compared different MOL levels of the same tissues in sika deer and identified 2927 and 107 DEGs in liver and kidney tissue, respectively.

After inspecting all the expanded/contracted gene families and DEGs in liver tissue, 29 genes were found to play roles in the P450 pathway. Of these, 3 were contracted genes, 20 were expanded genes from contracted or expanded gene families, and 12 were DEGs. The interaction network of these genes is shown in Figure 3D. Among these key genes, six *UGT2B4* and *UGT2B31* were both significantly up-regulated in high-tannin liver tissue and have multiple copies in the sika deer genome. The six up-regulation significantly differentially expressed *UGT* genes are distributed in chromosome 27, and three of these genes are related to gene duplication (Figure 3A and B). Therefore, we hypothesized that *UGT2B4* and *UGT2B31* are major genes in sika deer with high-tannin adaptation.

Interestingly, in liver tissue, tannins can drive the expression of many *UGT* genes in a dose-dependent manner. Overall, when compared among different MOL levels and ages (y0, y50, y100, and m100), eight differentially expressed *UGT* genes were discovered, among which two were down-regulated genes of the *UGT3A* subfamily and six were up-regulated genes in the *UGT2B* and *UGT2C* subfamilies (Figure 3E). Furthermore, we found that all of these up-regulated *UGT* genes in the liver were located on sika deer chromosome 27 (Figure 3F). With the increase in tannin content intake, the *UGT3A* subfamily genes in the liver were inhibited; nevertheless, *UGT* gene copies in the *UGT2B* and *UGT2C* families were increased, suggesting that the response of *UGT* gene expression to tannin was mainly up-regulated. Moreover, in the kidney tissue, two DEGs belonged to the *UGT2C* family. Five differentially expressed *CYP* genes were up-regulated, whereas gene families encoding glutathione S-transferase (*GST*) and sulfatyltransferase (*SULT*) were all down-regulated after the deer were fed a high-tannin diet.

According to previous studies, sika deer share common pathways with koala, including the drug metabolism-cytochrome P450 signal pathway [11]. The detoxification genes in sika deer showed different expression patterns compared with the genes in koala [11]. These results indicate that sika deer may utilize a different adaptive strategy from that of koala to survive on a diet of highly toxic food.

### High expression and expansion of the *UGT* genes contribute to tolerating a high-tannin diet

The sika deer diet of MOL contains high levels of tannins that would be lethal to most other mammals. Based on the aforementioned mechanism, genes involved in those pathways were examined using gene family and transcriptome analyses.

A total of 12 DEGs were detected from the *CYP2* subfamily in sika deer liver, but only 5 were differentially expressed with increasing tannin contents in the diet (Figure S14). Five *GST* genes and 3 *SULT* genes were found to be differentially expressed in the liver, but all were down-regulated with increasing tannin contents in the diet.

The functional importance of these *UGT* genes was further investigated through analysis of their expression levels in sika deer, showing that they had particularly high expression in the liver tissue, which is consistent with their role in detoxification. The mechanism of the glucuronidation reaction is that *UGT* enzymes catalyze the transfer of the glucuronosyl group from uridine 5′-diphospho-glucuronic acid (UDPGA) to the exogenous factor, generating the glucuronidated metabolite, which is more polar and more easily excreted than the exogenous factor (Figure 3F). Most of these expressed *UGT* genes belonged to *UGT2B*. These phenotypes suggest that *UGT* genes in *UGT2B* have an important role in detoxification; the up-regulated expansion of *UGT* genes would result in higher enzyme levels, which would enhance the ability of sika deer to detoxify the high-tannin diet.

Among the genes related to the metabolism of drugs and exogenous substances, *UGT* and *CYP* genes were found to be functionally involved in detoxification, especially *UGT* genes in the *UGT2B* family. In short, these findings imply that the unique expansion of the *UGT* gene family is mainly responsible for the toleration of high-tannin food, namely, oak leaves, by sika deer (Figure S15).

## Discussion

Cervidae is the second largest family in Artiodactyla [31] and has significant scientific [1] and economic [3] value. Although several other deer genomes have recently been reported, the lack of high-quality genome sequences of sika deer, one of the novel species in the family, has hindered the elucidation of the molecular mechanisms underlying important distinct biological characteristics of sika deer, such as the full regeneration of the antlers. Here, we sequenced the genome of sika deer and assembled it at the chromosome level using combined technologies of SMRT, Illumina sequencing, and Hi-C. The high percentage and accuracy rate of the genome structure, base calling, gene set validation, and quality of gene annotation demonstrated that our assembled sika deer genome was of high quality and could be effectively used as a reference genome for deer species.

The geographic distribution of sika deer is highly coincident with that of oak, and sika deer have a preference for grazing on high-tannin oak leaves [32], suggesting that this adaptation may be a positive selection during evolution. In terms of food adaption, sika deer are not unique. For example, pandas, dogs, and koalas have also undergone adaptive food evolution; pandas can eat bamboo despite being carnivorous [33], dogs can adapt to a diet of starchy foods [34], and koalas can eat toxic eucalyptus leaves [11]. Divergent adaptive pathways and related genes are known to be involved in this adaptation. In this study, we found that among the genes related to toxin degradation, only those from the *UGT* gene family [35], especially the *UGT2B* family, were significantly expanded. Furthermore, transcriptomic studies showed that *UGT* gene expression was strongly correlated with the quantity of tannin intake, and not all *UGT* genes show correlation, *i.e.*, it was dose dependent. The expression of specific extended gene copies in the *UGT2B* subfamily was prominently increased after the tannin feeding treatment and some of the up-regulation significantly differentially expressed *UGT* genes related to gene duplication. These results suggest that genes in the *UGT* family, especially the genes in the *UGT2B* subfamily, have a strong correlation with the adaptation of sika deer to a high-tannin diet.

It is generally believed that rumen microorganisms play a role in the digestion of tannins [36], [37]. However, as other ruminants, such as cattle and sheep, are not well adapted to high-tannin diets (Figure S16), we speculate that during a long period of coexistence with oak trees during evolution, sika deer may have developed genetic adaptive mechanisms. As expected, we found evidence for this phenomenon at the genome level through high-quality sequencing. Transcriptomic results also revealed that changes in gene expression were involved in Na and K ion channels. Based on Gene Ontology (GO), transcriptomic results also revealed that changes in gene expression were involved in the binding and transport of metal ions, for example, potassium ion transport (GO:0006813, BH-adjusted *P* < 0.05), ion transport (GO:0006811, BH-adjusted *P* < 0.05), and metal ion binding (GO:0046872, BH-adjusted *P* < 0.05). Interestingly, according to our social contact with Chinese farmers, they will feed oak leaves to stop the diarrhea of young deer. These genetic responses in Na^+^/K^+^ balance (water and salt metabolism) may enabled sika deer to adapt to oak leaves as an advantageous rather than a hazardous material for consumption. This interesting finding and the feeding habits of young sika deer suggest a future direction in which we could use young sika deer as a model for the study of diarrheaism.

The sika deer genome assembled in this study provides, to our knowledge, the highest quality deer genome to date. The comprehensive characterization of the sika deer genome along with the transcriptomic data presented herein provides a framework used to elucidate its evolutionary events, revealing the mechanism of the unique attributes and tannin adaptation. Through detailed genomics and transcriptomics analyses, we identified the most likely mechanism of tannin degradation in sika deer. We also depicted possible molecular mechanisms for the jungle adaptability of deer, and the methodologies we used in this study will also provide a reference for the study of the adaptation mechanism of animals to “toxic” foods. Chromosome-scale assembly of sika deer genomes could be used for many applications, including the study of structural variations in large genomic regions, expected recombination frequencies in specific genomic regions, target sequence characterization, and modification for gene editing. Moreover, this study provides a valuable genomic resource for research on the genetic basis of sika deer’s distinctive physiological features, such as the full regeneration of deer antlers, and on Cervidae genome evolution. Our study also contributes to conservation and utilization efforts for this antler-growing species.

## Materials and methods

### Sampling preparation

A female sika deer from Jilin Province was used for *de novo* genome sequencing. DNA was extracted from whole blood with a Genomic DNA Isolation Solution-type Kit (Catalog No. DP1102, BioTeke, Beijing, China) according to the manufacturer’s instructions. After slaughtering the experimental animals, tissue sampling was carried out immediately. Tissues, such as those from the hypothalamus, pituitary, gonad, liver, kidney, spleen, rumen, reticulum, and small intestine, were collected. RNA was extracted from the 15 tissue samples obtained from the animals. After library construction and size selection, 150.4 Gb (∼ 57.7×) of long reads with a mean length of 9205 bp were generated by the PacBio RSII platform. In addition, 261.5 Gb (∼ 100.6×) of paired-end data with varying insert sizes (200 bp, 300 bp, 400 bp, and 600 bp) were generated by the Illumina HiSeq 2000 platform (Figure S17).

### *De novo* genome sequencing and Hi-C-based assembly

The PacBio subreads were used to perform *de novo* genome assembly via wtdbg (v1.2.8) [17] with the key parameter “-H -k 19”. Then, primary assemblies were polished using the Quiver [38] algorithm with the default parameters. A total of 93.4× clean paired-end reads from the Illumina platform were aligned to the Quiver-polished assemblies using BWA (v0.7.10-r943-dirty) with default parameters to reduce the remaining indel and base substitution errors in the draft assembly. Inconsistent sequences between the polished genome and Illumina reads were identified with SAMtools/BCFtools (v1.3.1) with default parameters. The credible homozygous variations with differences in quality exceeding 20, a mapping quality greater than 40, and a sum of high-quality alt-forward and alt-reverse bases more than 2 in the Quiver-polished assemblies were replaced by the called bases using in-house scripts (see Code availability). Finally, highly accurate contigs were generated.

Four billion PE150 reads were produced from three Hi-C libraries by the Illumina HiSeq platform. Hi-C-based proximity-guided scaffolding was used to connect primary contigs. Clean reads were first aligned against the reference genome with the Bowtie2 end-to-end algorithm. HiC-Pro (v2.7.8) with “LIGATION_SITE = AAGCTAGCTT” and other default parameters was then able to detect the ligation sites and align them back to the genome with the 5′ fraction of the reads. The assembly tool LACHESIS was applied for clustering, ordering, and orienting. Based on the agglomerative hierarchical clustering algorithm, we clustered the contigs into 33 groups. For each chromosome cluster, we obtained an exact scaffold order of the internal groups and traversed all the directions of the scaffolds through a weighted directed acyclic graph (WDAG) to predict the orientation for each scaffold. A chromosome-scale assembly with 33 clusters was obtained that anchored 99.24% of the contigs for sika deer.

### Genome accuracy assessment

To determine the completeness and accuracy of the MHL_v1.0 assembly, we carried out the following validation. First, the MHL_v1.0 assembly was aligned to the red deer genome (CerEla1.0) and BioNano optical maps. The conflicting regions that appeared in both alignments were potential misassemblies and were manually inspected and corrected.

A total of 2715 EST sequences of sika deer were downloaded from the NCBI dbEST database and aligned with MHL_v1.0 using BLAST (v35). The BUSCO (v3.1.0, OrthoDB v9) [39] software package was used to assess the quality of the generated genome using the genome model “- M genome”. The CEGMA (v2.5) [19] pipeline software with parameter “--mam”, was also run against the MHL_v1.0. Illumina short reads (∼ 93.4×), was aligned to MHL_v1.0 with BWA to estimate the accuracy of a single base of the assembly, which was based on the count of homozygous single nucleotide polymorphisms (SNPs).

### Repeat sequence annotation

To annotate the sika deer genome, RepeatModeler (v1.0.8) with default parameters was initially used to obtain a *de novo* repeat library. Next, RepeatMasker (v4.0.5) was used to search for known and novel TEs by mapping sequences against the Repbase TE library (20150807) [40] by using parameters “-s -xsmall”.

### Gene annotation

For *de novo* gene prediction, we utilized Augustus (v3.0.3), SNAP (v2006-07-28), GlimmerHMM (v3.0.4), and GENSCAN to analyze the repeat-masked genome. For homology-based gene predictions, the protein sequences of human, mouse, cattle, sheep, and horse were mapped to the sika deer genome with GenBlastA [41]. Then, the prediction was performed with GeneWise (v2.2.3) [42] in aligned regions. RNA-seq reads were aligned to the genome using TopHat (v2.0.12) and assembled by Cufflinks (v 2.2.1) with the default parameters. EVidenceModeler software (EVM, v1.1.1) was used to integrate the genes predicted by homology, *de novo,* and transcriptome approaches and generate a consensus gene set. Short-length (<50 aa) and transcriptome data for nonsupport genes were removed from the consensus gene set, and the final gene set was produced.

We translated the final predicted coding regions into protein sequences and mapped all the predicted proteins to the Swiss-Prot, TrEMBL, and Kyoto Encyclopedia of Genes and Genomes (KEGG) databases using BLASTP (v2.2.27+) for gene functional annotation. We used the InterProScan (v5.21-60.0) database to annotate the motifs, domains, and GO terms of proteins with retrieval from the Pfam, PRINTS, PROSITE, ProDom, and SMART databases.

### Gene family construction

Annotations of human, mouse, pig, sheep, and cattle genomes were downloaded from Ensembl (release-87), while those of minke whale, dromedary, Bactrian camel, yak, goat, white-tailed deer, red deer, and reindeer were downloaded from NCBI. To annotate the structures and functions of putative genes in the giraffe, okapi, milu, musk deer, and roe deer assemblies, we used homology-based predictions. Cattle proteins (Ensemble release-87) were aligned to the 5 genomes using GenBlastA (v1.0.1) [41] and predicted by GeneWise (v2.2.3) [42]. The genes of the above 18 species and sika deer were used to construct gene families using TreeFam [17]. All the protein sequences were searched in the TreeFam (version 9) HMM file and classified among different TreeFamilies.

### Phylogeny and divergence time estimation

Sika deer and 18 other mammalian taxa (human, mouse, pig, sheep, cattle, minke whale, dromedary, Bactrian camel, yak, goat, white-tailed deer, red deer, reindeer, giraffe, okapi, milu, musk deer, and roe deer) were used in the phylogenetic analysis. The protein sequences of these 19 species were mapped to TreeFam (version9) [17] using hmmsearch with the parameters -noali -max -Z 1000 to get the orthologous genes, and 748 single-copy orthologous genes were determined. Multiple sequence alignments of these 748 genes were calculated by MUSCLE [43] software, and were combined into a long sequence for each species. Then, the conserved block regions of the alignment were picked out by Gblocks 0.91b [44] with default parameters. With the input of this alignment, phylogenetic tree was constructed by RAxML (v8.2.9) [45] software with GTRGAMMA model and bootstrap 1000. Divergence times were estimated by Phylogenetic Analysis by Maximum Likelihood (PAML, v4.8a) [46] mcmctree. The Markov chain Monte Carlo (MCMC) process was run for 20,000 iterations with a sample frequency of 2 after a burn-in of 1000 iterations. Other parameters used the default settings of mcmctree. Two independent runs were performed to check convergence. The following constraints were used for fossil time calibrations: (1) Bovinae and Caprinae divergence time (18–22 MYA); (2) Ruminantia and Suina divergence time (48.3–53.5 MYA); (3) Euarchontoglires and Laurasiatheria divergence time (95.3–113 MYA); (4) Euarchontoglires and Rodentia divergence time (85–94 MYA); and (5) *Cervus* and *Elaphurus* divergence time (<3 MYA).

### Gene family expansions and contractions

The CAFE program (v3.1) [47] was used to analyze gene family expansions and contractions. The program uses a birth and death process to model gene gain and loss across a user-specified phylogenetic tree. The numbers of sika deer genes relative to the number of inferred ancestor genes and expanding and contracting gene families were obtained. The function “phyper” in R software was used to conduct the enrichment analyses on the expanded and contracted gene families obtained by CAFE. The hypergeometric test was used to calculate enrichment KEGG pathways and Go terms depending on the number of genes that are both in expanded/contracted gene families and the pathway, and the number of all genes in the pathway. To control the false discovery rate (FDR), *P* value was corrected by BH method by R command “p.adjust” with parameter “method = BH”. A *P* value less than 0.05 after the correction was considered a significant enrichment result.

We investigated several *UGT* genes in each category for the 19 species. The annotated *UGT* genes of human and sika deer were used to predict the unannotated *UGT* genes in the other 17 species with the program GeneWise [42]. MUSCLE software was used for the multiple sequence alignment of all these *UGT* gene protein sequences, whereby a phylogenetic *UGT* gene tree was constructed using RAxML [45].

### Synteny analysis

A collinearity analysis between sika deer and red deer was conducted using the MUMmer package (v3.23). Furthermore, to identify the synteny block among sika deer, red deer, cattle, and goat, we used MCScan (python version) [48] to search for and visualize intragenomic syntenic regions. A homologous synteny block map between sika deer and cattle was plotted with Circos.

### Demographic history reconstruction

We inferred the demographic histories of sika deer and red deer using the PSMC model for diploid genome sequences. We downloaded the sequencing data (SRR4013902) of red deer in NCBI. 242.9 Gb short reads of sika deer (∼ 93.4×) and 222.8 Gb short reads of red deer (∼ 64.8×) were mapped to the sika genome (MHL_v1.0) and red deer genome (CerEla1.0) with BWA (v0.7.10-r943-dirty) respectively, then the diploid consensus sequence was generated by SAMtools. Program “fq2psmcfa” in PSMC transforms the consensus sequence into a fasta-like format as the input for PSMC. The parameters for “psmc” were set as follows: -N25 -t15 -r5 -p “4 + 25×2 + 4 + 6”. The generation times (g) of sika deer and red deer were both 5 years, respectively. The mutation rate for all species was 2.0E–9 per site per year [13] and the per generation mutation rate was 1.0 E–8, calculated by multiplied the per year mutation rate by the generation length.

### PSGs

Multiple sequence alignment was carried out using MUSCLE (v3.8.31) for the single-copy orthologous genes of 19 species. Regions of uncertain alignment were removed by Gblocks 0.91b [49]. We used branch-site models and likelihood ratio tests (LRTs) in the CODEML of PAML (v4.8a) [46] to detect PSGs in the sika deer genome. *P* values were computed using the χ^2^ statistic and corrected for multiple testing by the FDR method (BH-adjusted *P* < 0.05). All the PSGs were mapped to KEGG pathways and assigned GO terms. GO and KEGG enrichment analyses were then applied to detect the significantly enriched biological processes and signaling pathways of PSGs (BH-adjusted *P* < 0.05).

### Transcriptome analysis

We performed RNA sequencing of 15 tissues (hypothalamus, liver, muscle, spleen, kidney, testis, pituitary, appendix, duodenum, ileum, jejunum, rumen, abomasum, reticulum, and omasum) for each of the 12 sika deer from the feeding trials to determine variations in gene expression levels after treatment. To compare the response to different tannin levels between cattle and sika deer, we conducted RNA-seq and transcriptome analyses of 8 tissues (hypothalamus, liver, kidney, rumen, jejunum, pituitary, reticulum, and spleen) from two groups of 6 individuals with a diet containing 0% or 10% gallotannic acid (GA). The high quality total RNA was isolated from the 15 tissues using TRIzol Reagent (Catalog No. 15596-018, Invitrogen life Technologies, Carlsbad, CA) according to the manufacturer’s instructions. The purity of RNA was determined using a Nanodrop 2000 spectrophotometer (Catalog No. NANODROP 2000, ThermoFisher Scientific, Waltham, MA), the concentration of RNA was measured using Qubit 2.0 fluorometer (Catalog No. Q32866, ThermoFisher Scientific), and the RNA integrity was determined using Agilent 2100 Bioanalyzer (Catalog No. G2939A, Agilent, Palo Alto, CA). Total RNA from 226 feeding experiment samples was extracted and used for library construction and sequencing. All libraries were sequenced using an Illumina HiSeq platform.

The transcriptome data of each sample were mapped to the sika deer and cattle genomes using HISAT2 (v2.0.5) with parameter “--dta”, and gene expression was calculated in each sample using StringTie (v1.3.0) with default parameters. The R language package DESeq2 was used to homogenize the expression and calculate the pairwise differential expression between samples with different treatment conditions under the same tissue, in which genes with *P* adjusted <0.05 were considered DEGs. For the DEGs, the hypergeometric test and BH algorithm were used in the GO and KEGG enrichment analysis and *P* value correction, respectively. A Q value <0.05 was considered significantly enriched in the GO and KEGG pathways.

## Ethical statement

All procedures concerning animals were performed in accordance with the guidelines for the care and use of experimental animals established by the Ministry of Agriculture and Rural Affairs of China, and all protocols were approved by the Institutional Animal Care and Use Committee of Institute of Special Economic Animal and Plant Sciences, Chinese Academy of Agricultural Sciences (Approval No. ISAPSAEC-2014-016), Changchun, China.

## Code availability

The in-house script “vcf_revise_ctg”, used to correct single bases and small indel errors, has been deposited in the Biocode at the National Genomics Data Center (NGDC), Beijing Institute of Genomics (BIG), Chinese Academy of Sciences (CAS)/China National Center for Bioinformation (CNCB) (Biocode: BT007282), and is publicly accessible at https://ngdc.cncb.ac.cn/biocode. Please read the manual page for detailed installation and usage.

## Data availability

The whole-genome sequence data reported in this study have been deposited in the Genome Warehouse (GWH) [50] at the NGDC, BIG, CAS / CNCB (GWH: GWHANOY00000000), which are publicly accessible at https://ngdc.cncb.ac.cn/gwh. The raw sequence data have been deposited in the Genome Sequence Archive (GSA) [51] at the NGDC, BIG, CAS / CNCB (GSA: CRA001393, CRA002054, and CRA002056), which are publicly accessible at https://ngdc.cncb.ac.cn/gsa.

## Competing interests

Wei Zhan is a current employee of Annoroad Gene Technology (Beijing) Co., Ltd. Qiqi Liang is a former employee of Novogene Co. Ltd. All the other authors have declared no competing interests.

### CRediT authorship contribution statement

**Xiumei Xing:** Funding acquisition, Conceptualization, Supervision, Project administration. **Cheng Ai:** Writing – review & editing, Formal analysis, Visualization, Methodology, Data curation, Software. **Tianjiao Wang:** Writing – review & editing, Formal analysis, Visualization, Software. **Yang Li:** Writing – review & editing, Formal analysis, Visualization. **Huitao Liu:** Writing – review & editing, Investigation, Validation. **Pengfei Hu:** Writing – original draft. **Guiwu Wang:** Investigation, Validation. **Huamiao Liu:** Resources. **Hongliang Wang:** Resources. **Ranran Zhang:** Resources. **Junjun Zheng:** Investigation, Validation. **Xiaobo Wang:** Formal analysis, Software. **Lei Wang:** Resources. **Yuxiao Chang:** Resources. **Qian Qian:** Writing – review & editing. **Jinghua Yu:** Data curation. **Lixin Tang:** Resources. **Shigang Wu:** Software. **Xiujuan Shao:** Formal analysis, Software. **Alun Li:** Software. **Peng Cui:** Resources. **Wei Zhan:** Software. **Sheng Zhao:** Resources. **Zhichao Wu:** Software. **Xiqun Shao:** Resources. **Yimeng Dong:** Resources. **Min Rong:** Resources. **Yihong Tan:** Data curation. **Xuezhe Cui:** Resources. **Shuzhuo Chang:** Resources. **Xingchao Song:** Resources. **Tongao Yang:** Resources. **Limin Sun:** Resources. **Yan Ju:** Resources. **Pei Zhao:** Resources. **Huanhuan Fan:** Resources. **Ying Liu:** Resources. **Xinhui Wang:** Resources. **Wanyun Yang:** Resources. **Min Yang:** Resources. **Tao Wei:** Resources. **Shanshan Song:** Resources. **Jiaping Xu:** Resources. **Zhigang Yue:** Resources. **Qiqi Liang:** Writing – review & editing. **Chunyi Li:** Funding acquisition, Conceptualization, Supervision, Project administration. **Jue Ruan:** Conceptualization, Supervision, Project administration, Methodology, Data curation. **Fuhe Yang:** Funding acquisition, Conceptualization, Supervision, Project administration. All authors have read and approved the final manuscript.
