# Supplementary figures and images for: The First High-quality Reference Genome of Sika Deer Provides Insights into High-tannin Adaptation
Source: Genomics Proteomics Bioinformatics. 2022 Jun 16;21(1):203–15. doi: 10.1016/j.gpb.2022.05.008 (PMC10372904; doi:10.1016/j.gpb.2022.05.008)

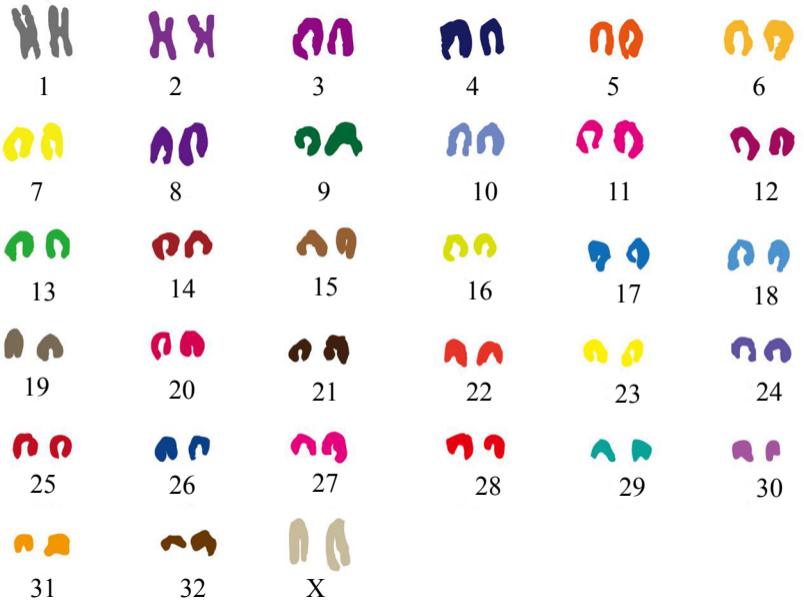

Supplement: Supplementary Figure S1 [file mmc1.pdf]

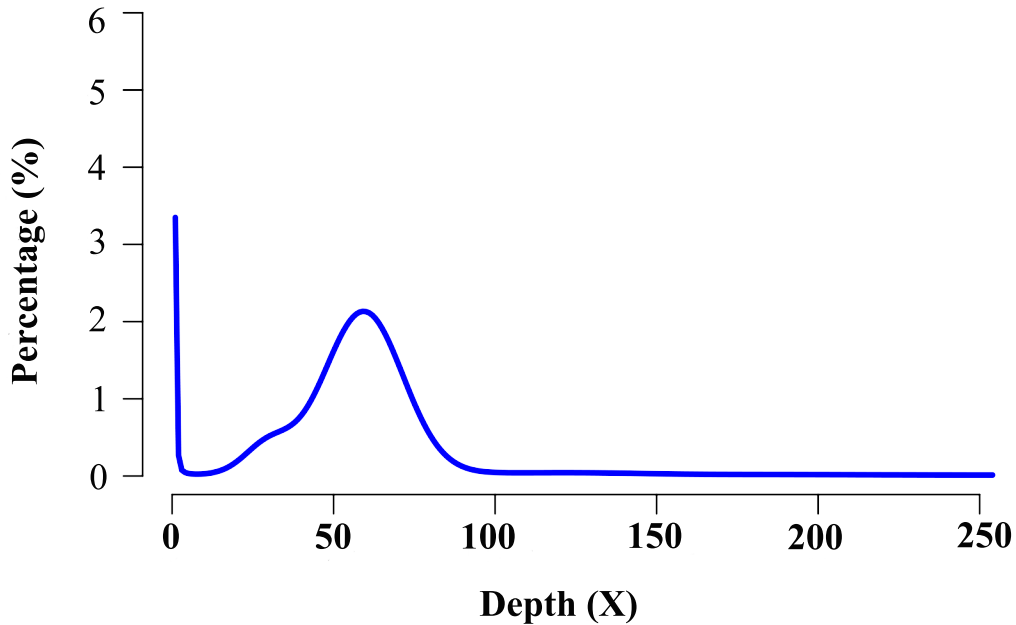

Supplement: Supplementary Figure S2 [file mmc2.pdf]

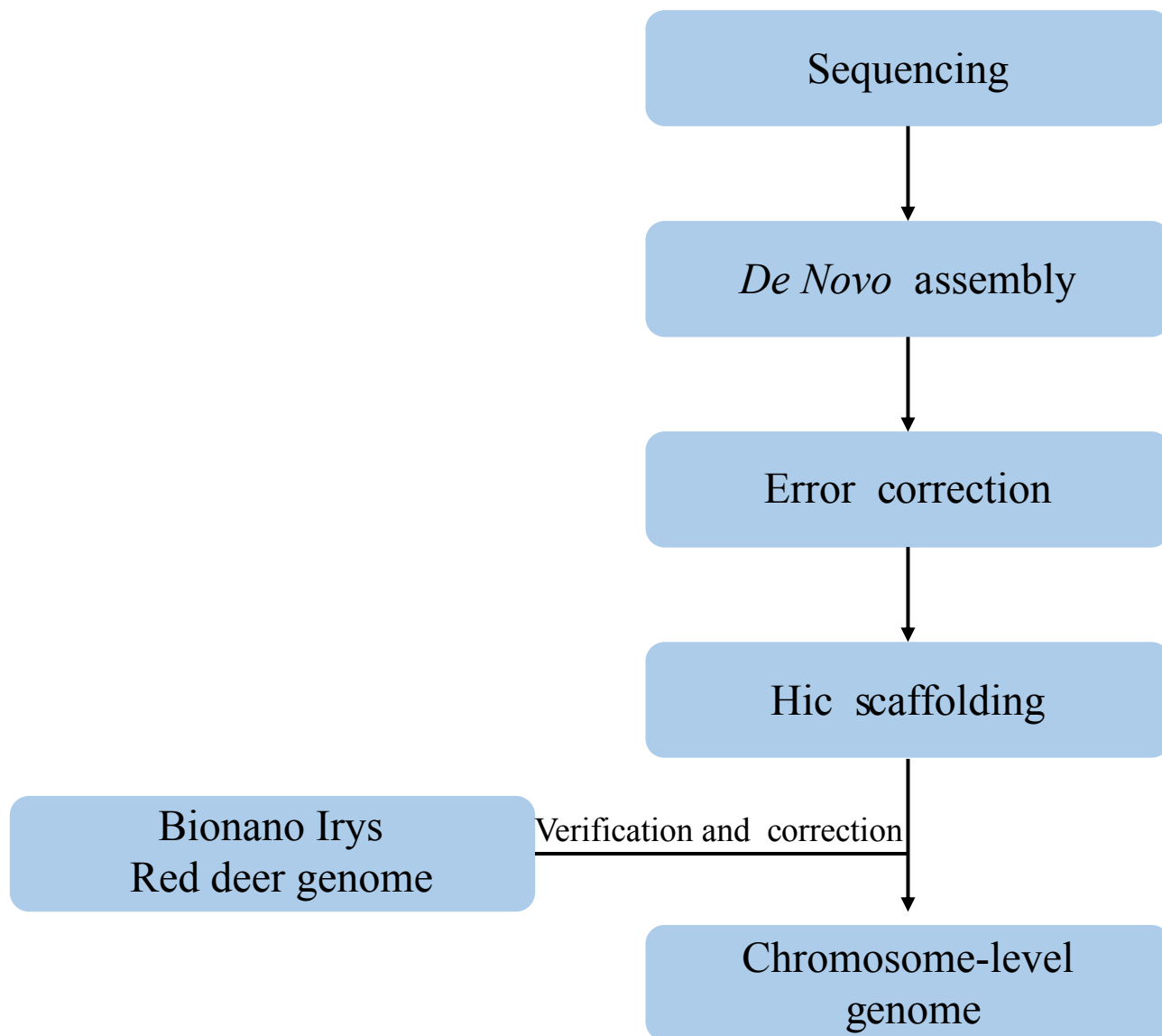

Supplement: Supplementary Figure S3 [file mmc3.pdf]

Sika deer

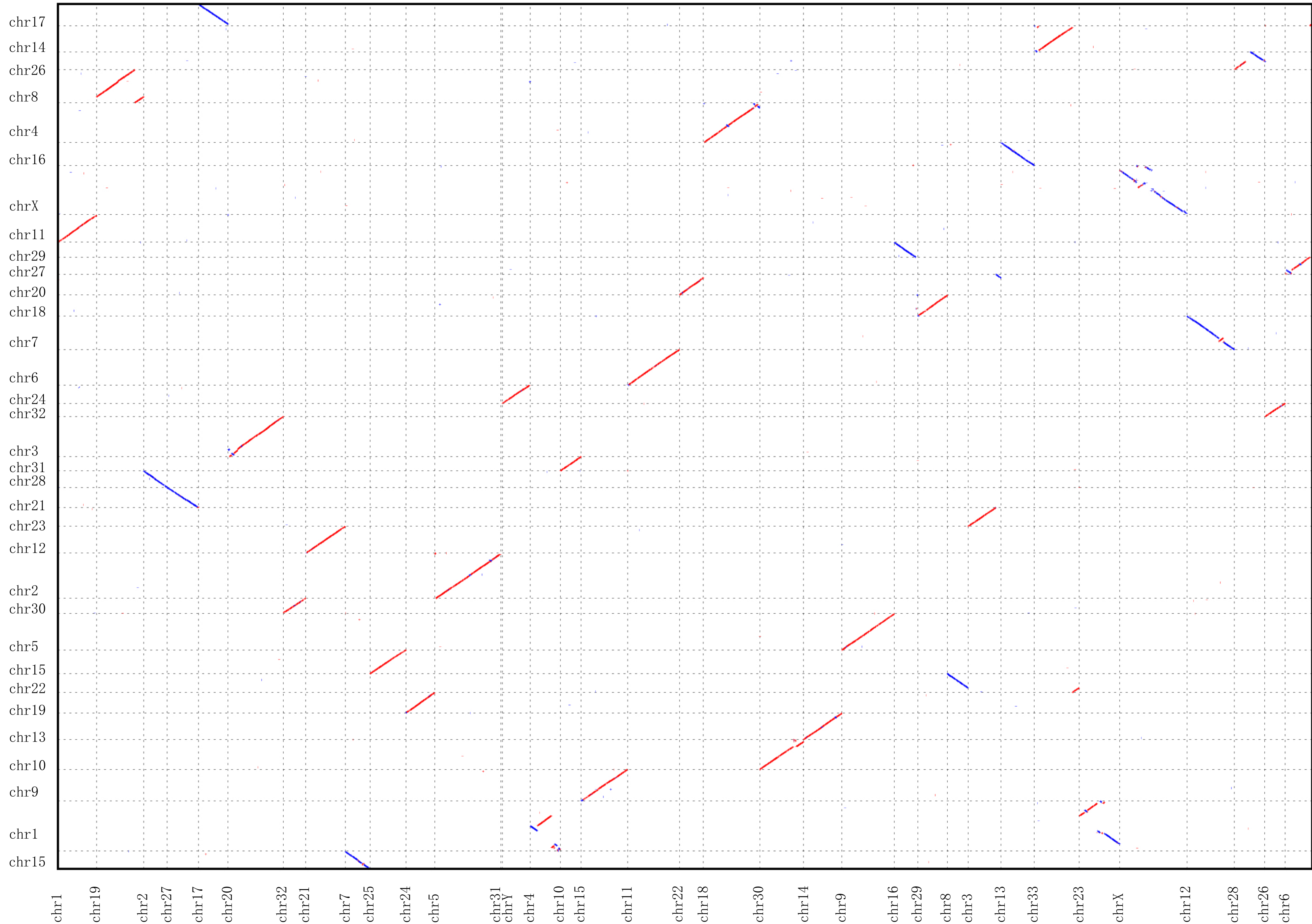

Supplement: Supplementary Figure S4 [file mmc4.pdf]

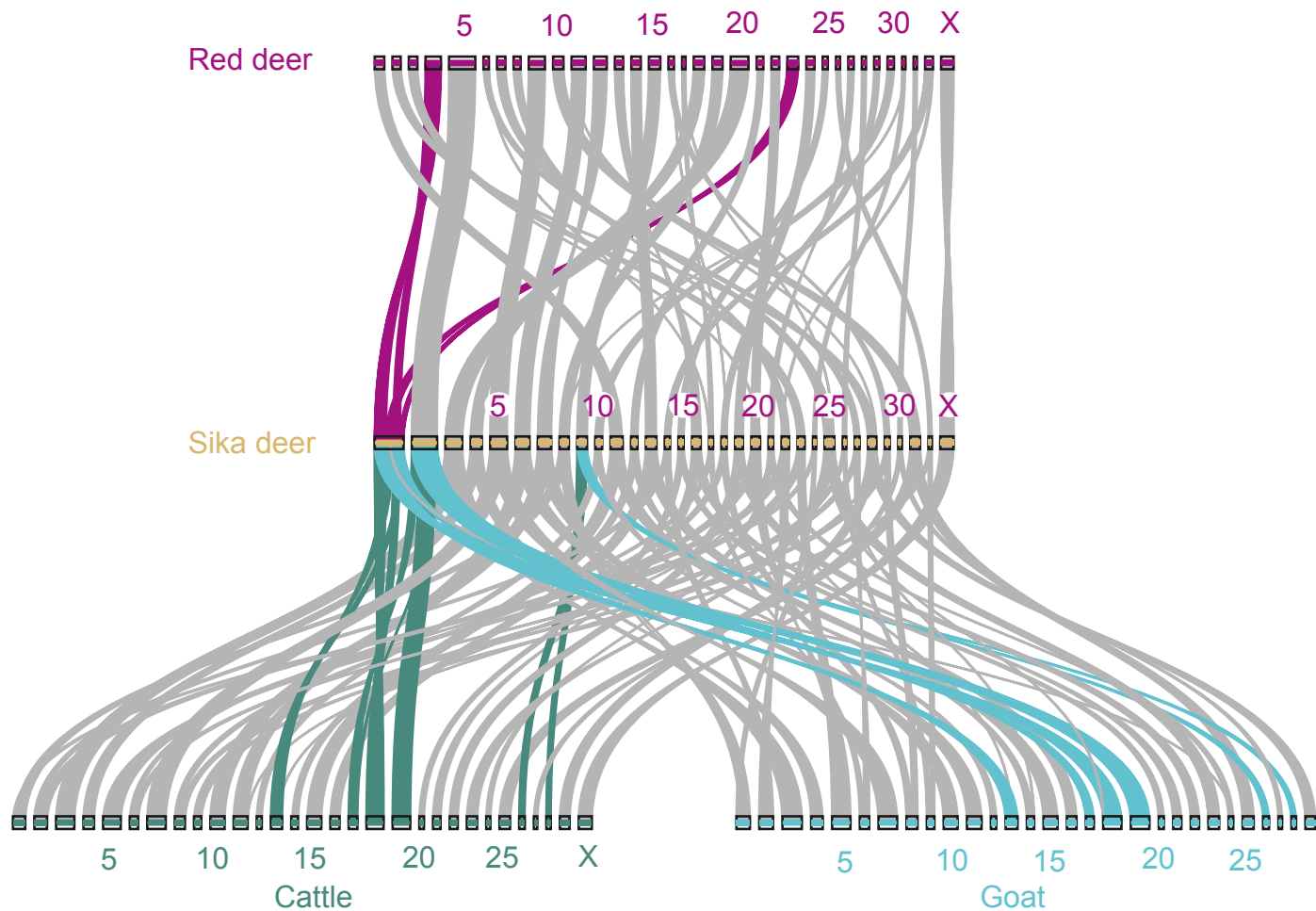

Supplement: Supplementary Figure S6 [file mmc6.pdf]

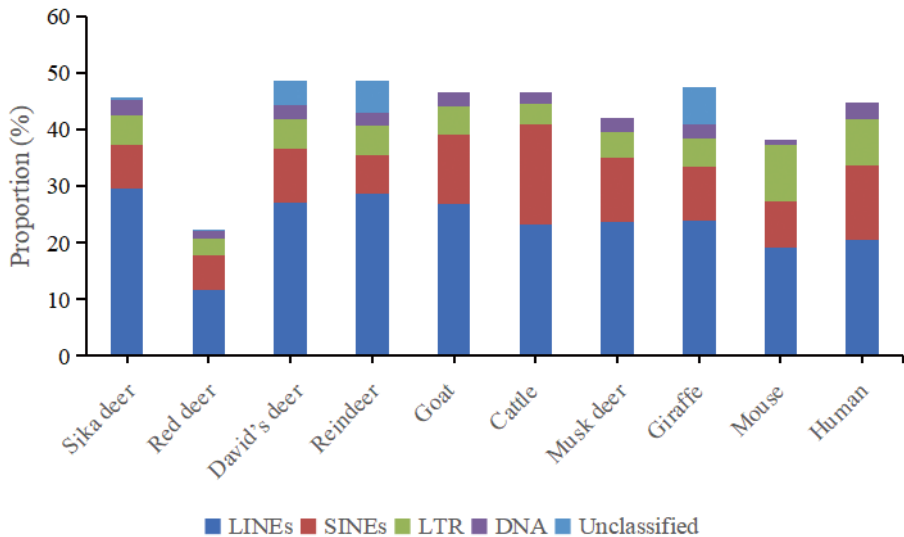

Supplement: Supplementary Figure S7 [file mmc7.pdf]

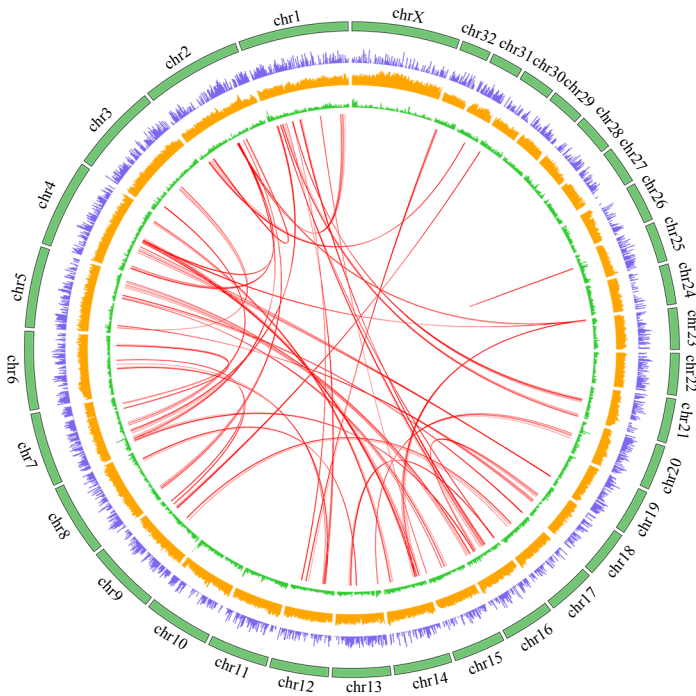

Supplement: Supplementary Figure S8 [file mmc8.pdf]

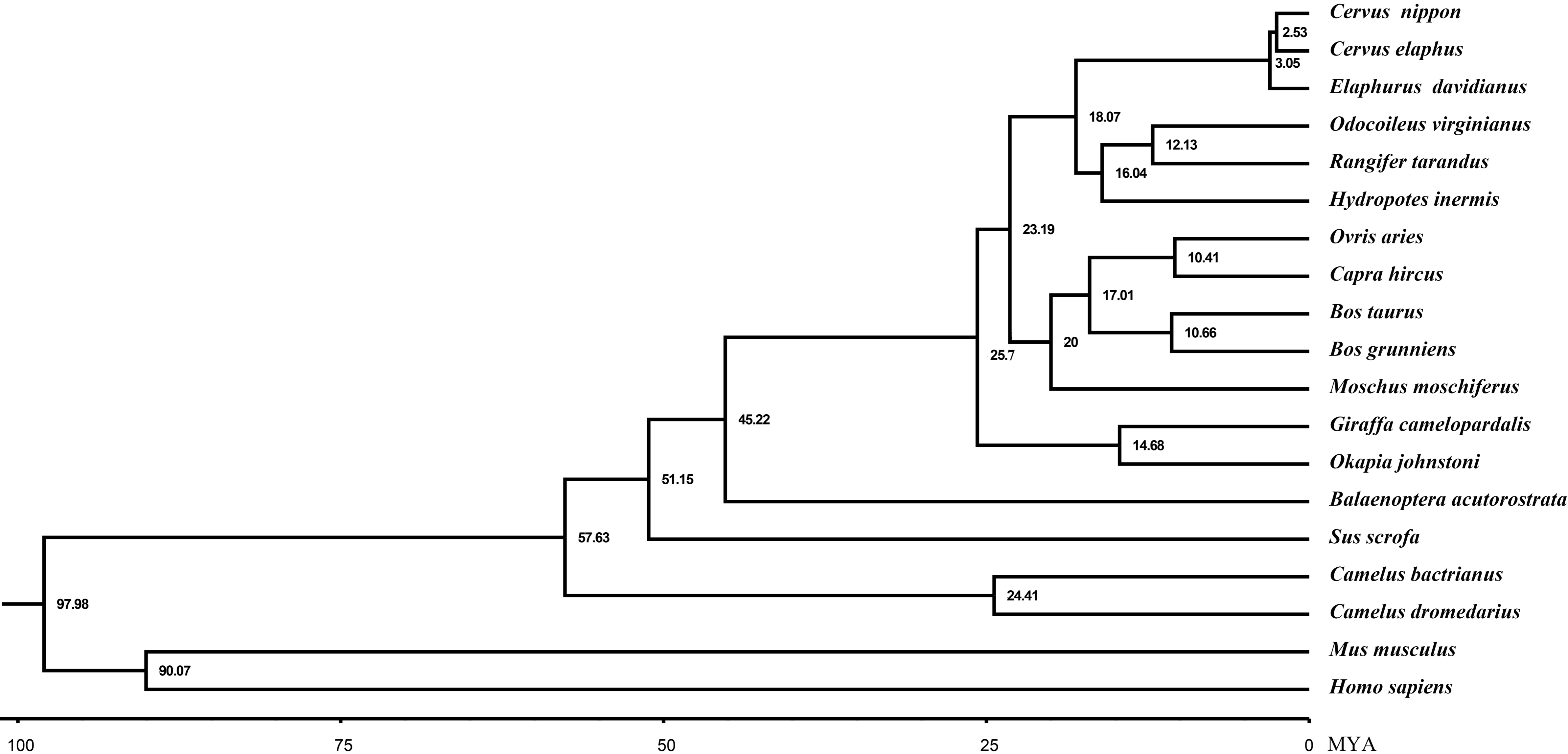

Supplement: Supplementary Figure S9 [file mmc9.pdf]

+/- Gene families gain/loss

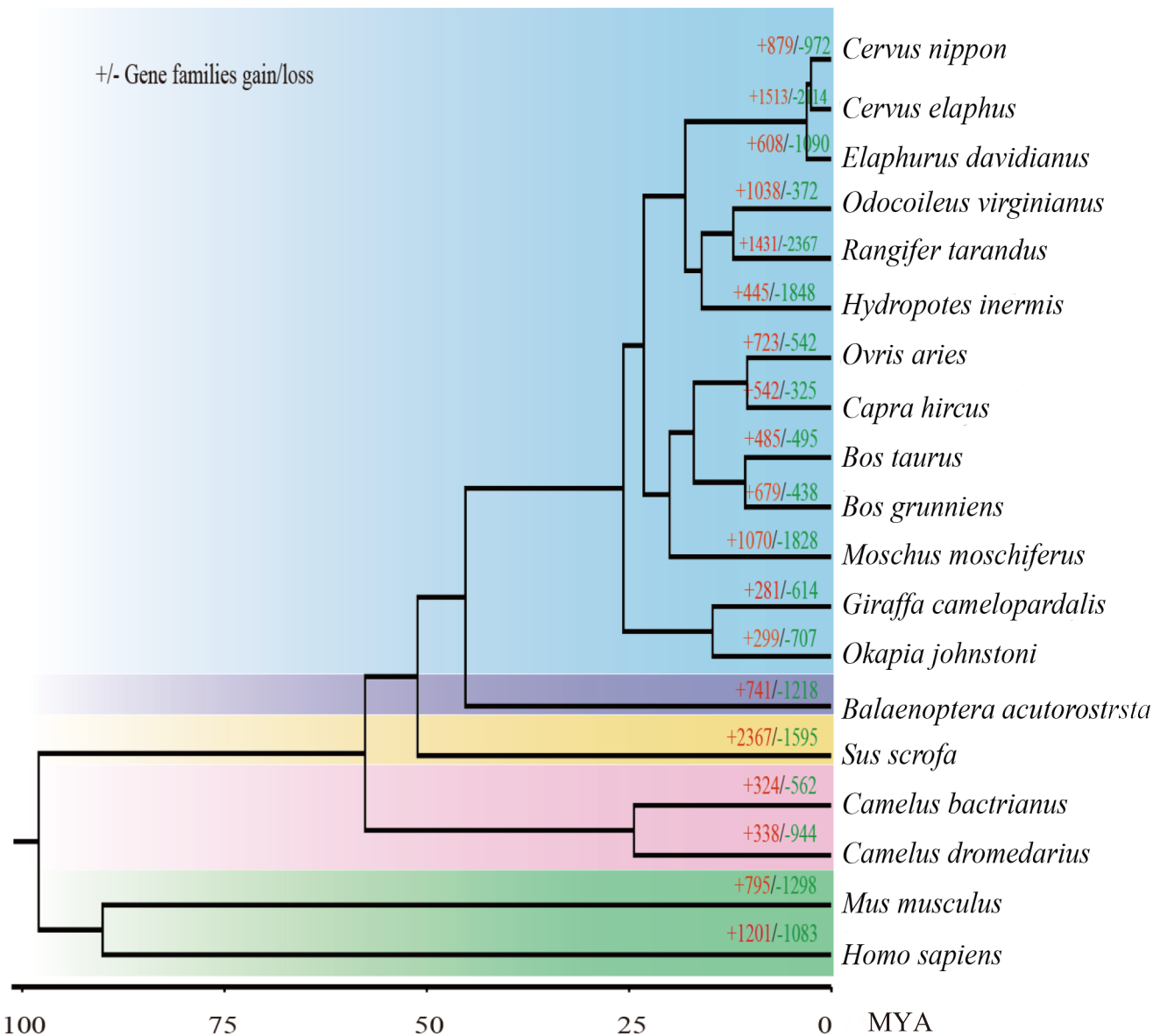

Supplement: Supplementary Figure S10 [file mmc10.pdf]

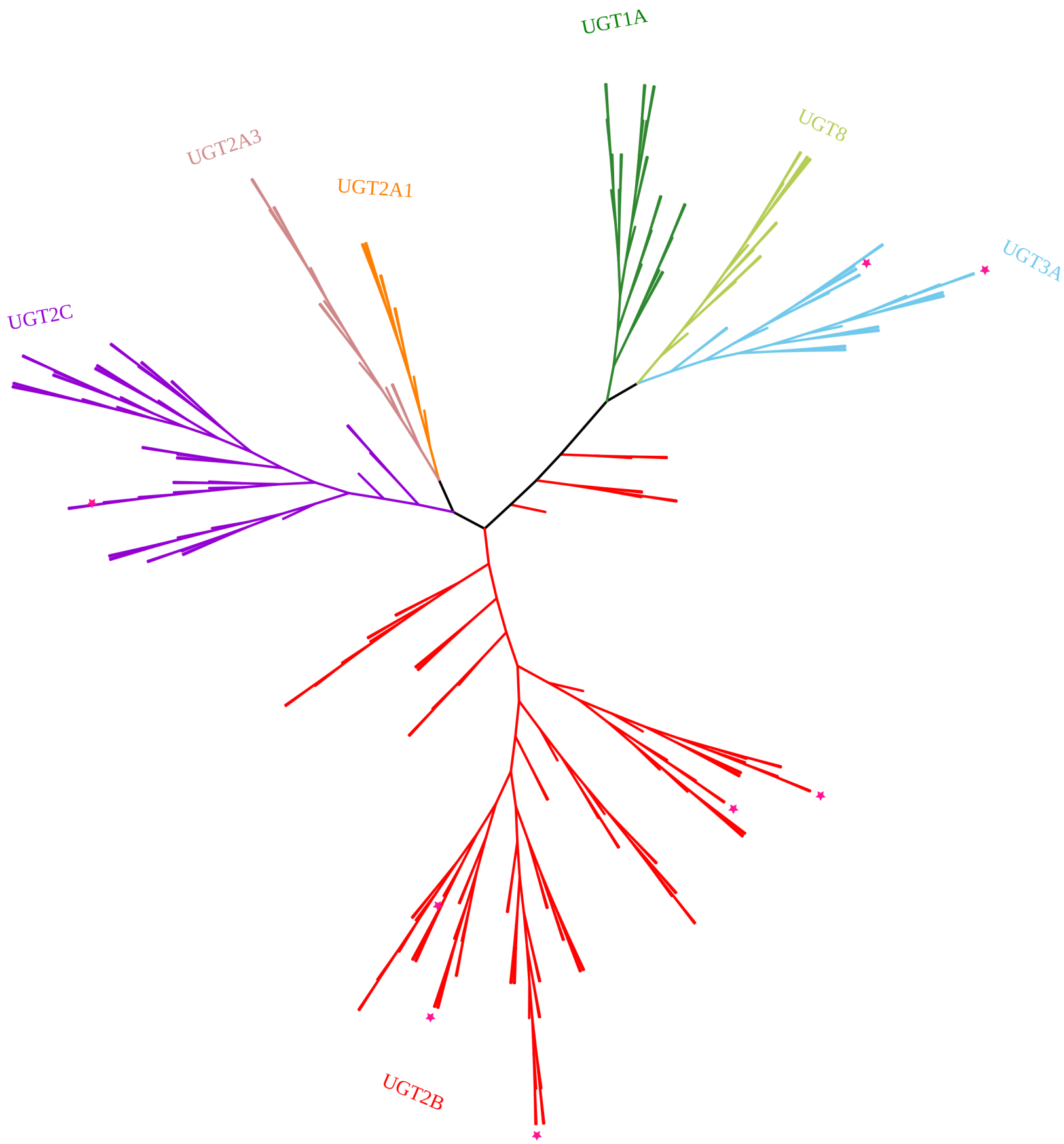

Supplement: Supplementary Figure S11 [file mmc11.pdf]

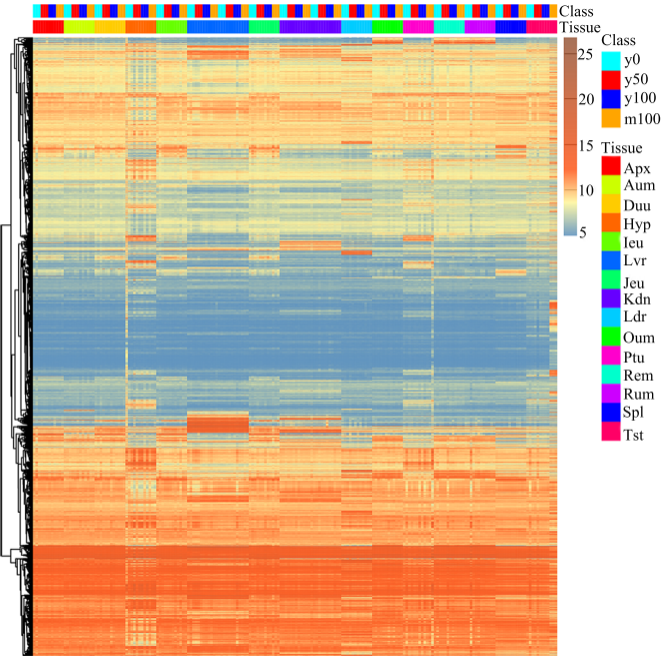

Supplement: Supplementary Figure S12 [file mmc12.pdf]

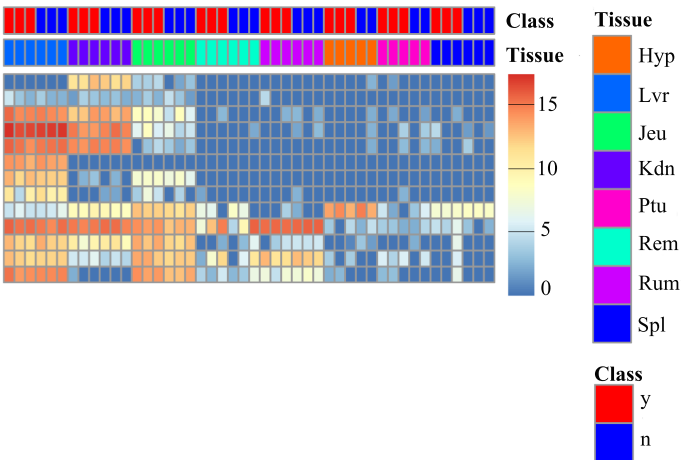

Supplement: Supplementary Figure S13 [file mmc13.pdf]

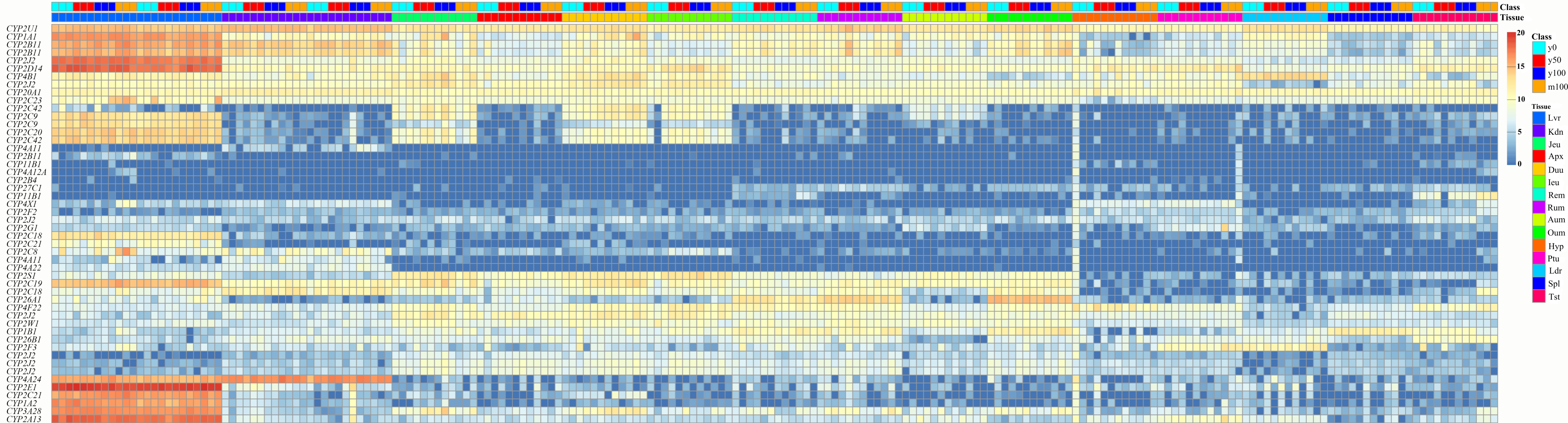

Supplement: Supplementary Figure S14 [file mmc14.pdf]

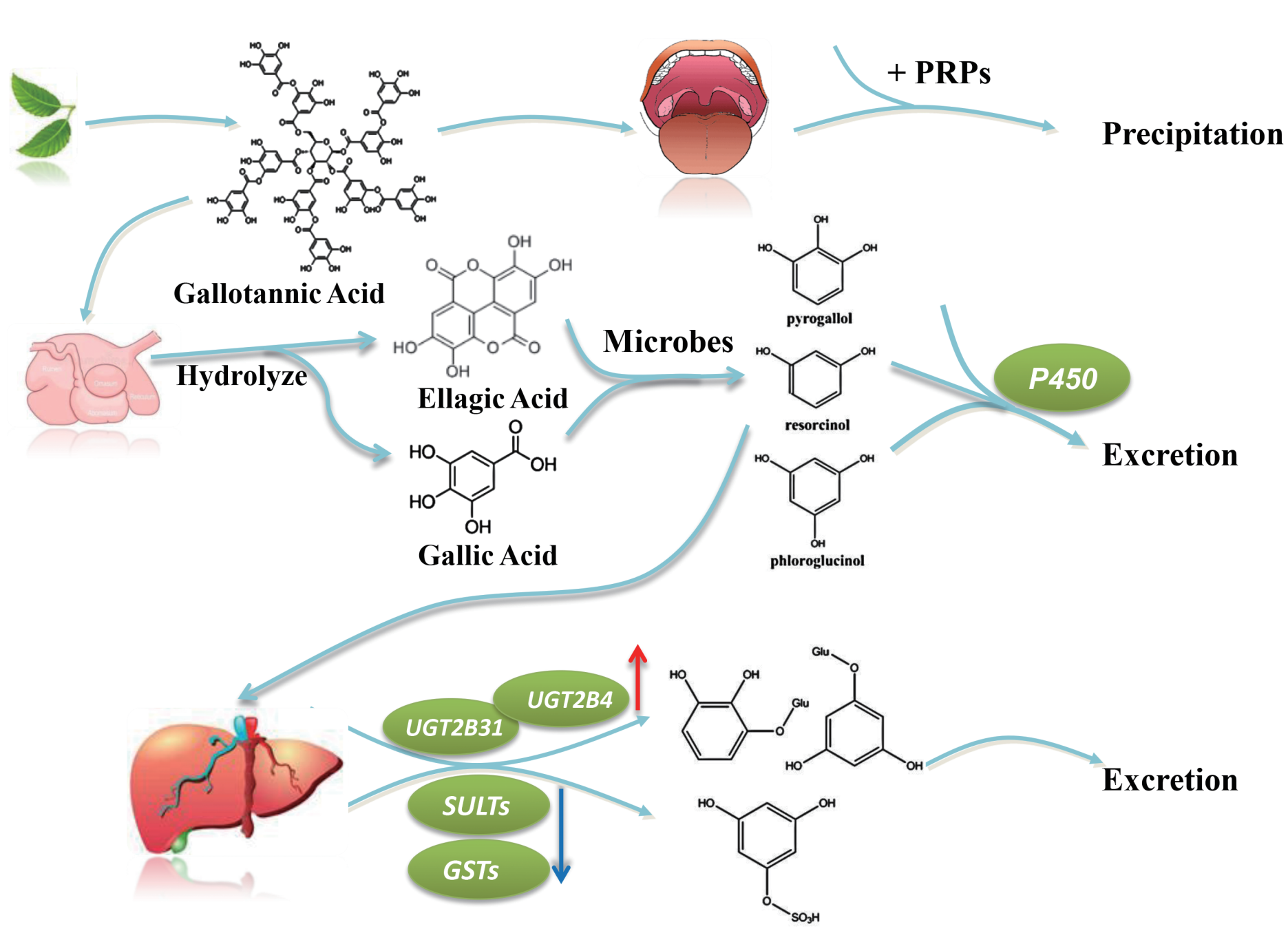

Supplement: Supplementary Figure S15 [file mmc15.pdf]

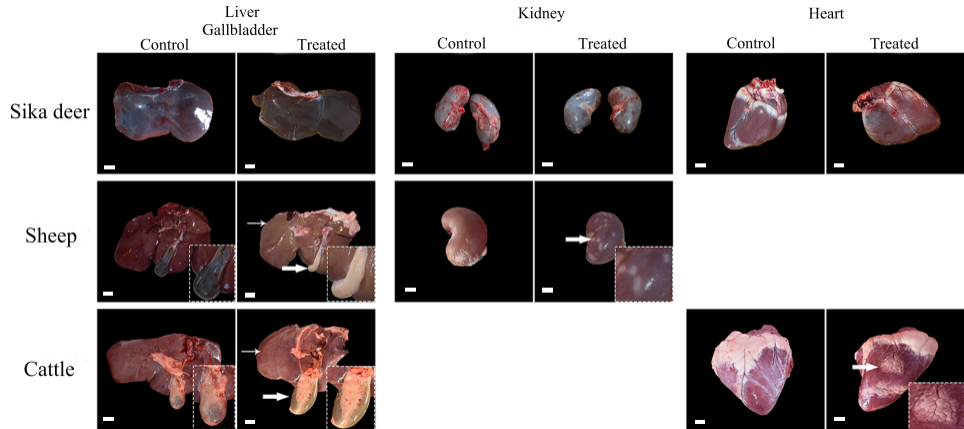

Supplement: Supplementary Figure S16 [file mmc16.pdf]

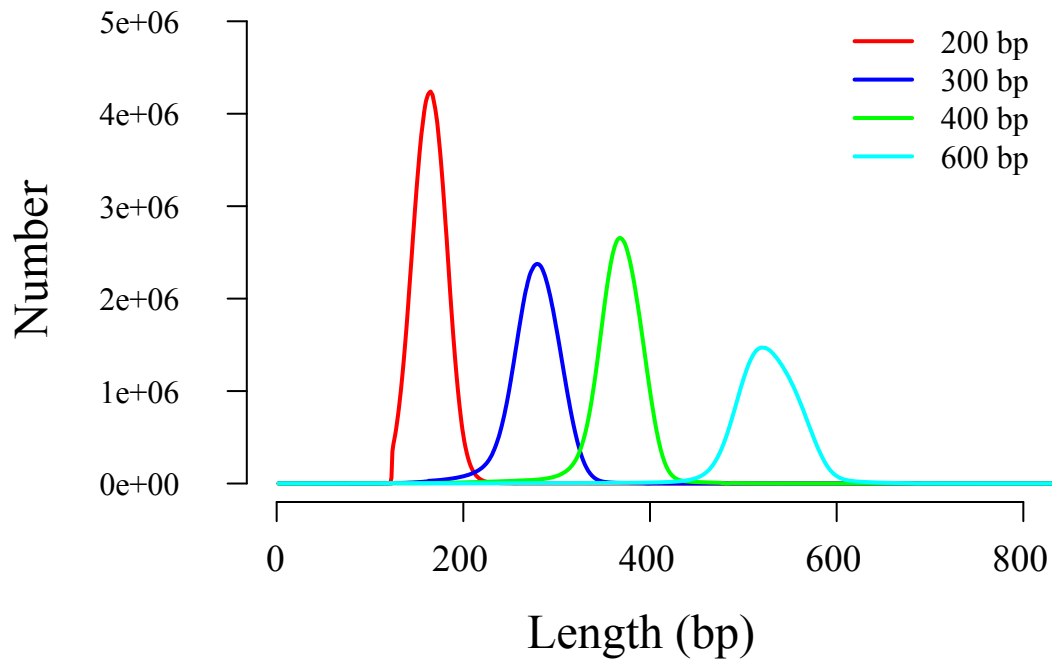

Supplement: Supplementary Figure S17 [file mmc17.pdf]
