# Supplementary material for: The First High-quality Reference Genome of Sika Deer Provides Insights into High-tannin Adaptation
Source: Genomics Proteomics Bioinformatics. 2022 Jun 16;21(1):203–15. doi: 10.1016/j.gpb.2022.05.008 (PMC10372904; doi:10.1016/j.gpb.2022.05.008)
Supplement: Supplementary Table S1 [file mmc18.docx]

**Table S1**  **Estimation of the sika deer genome size using K-mer analysis**

| Species | K-mer length | Read length | Error ratio (%) | K-mer number (M) | Uniq K-mer number (M) | Repeat ratio (%) | First peak | Genome size (Mb) | Data size (Mb) | K-mer depth | Coverage depth |
| --- | --- | --- | --- | --- | --- | --- | --- | --- | --- | --- | --- |
| Sika deer | 25 | 125 | 0.155 | 154,474 | 7764 | 11.9 | 59 | 2614.17 | 244,644.28 | 59.09 | 93.58 |
