# Supplementary material for: The First High-quality Reference Genome of Sika Deer Provides Insights into High-tannin Adaptation
Source: Genomics Proteomics Bioinformatics. 2022 Jun 16;21(1):203–15. doi: 10.1016/j.gpb.2022.05.008 (PMC10372904; doi:10.1016/j.gpb.2022.05.008)
Supplement: Supplementary Table S2 [file mmc19.docx]

**Table S2 Summary of the genome sequencing of sika deer**

| **Platform** | **Insert size** | **Read**  **length (bp)** | **Raw data** | | **Qualified data** | |
| --- | --- | --- | --- | --- | --- | --- |
|  |  |  | **Total data (G)** | **Sequence**  **coverage^1^** | **Total data (G)** | **Sequence coverage^1^** |
| Illumina | 200 bp | 125 bp | 66.8 | 25.7 | 65.1 | 25.0 |
|  | 300 bp | 125 bp | 64.6 | 24.8 | 60.5 | 23.3 |
|  | 400 bp | 125 bp | 67.2 | 25.8 | 65.3 | 25.1 |
|  | 600 bp | 125 bp | 62.9 | 24.2 | 52.0 | 20 |
|  | Total |  | 261.5 | 100.6 | 242.9 | 93.4 |
| PacBio |  |  | 150.4 | 57.7 |  |  |

*Note*: ^1^ K-mer analysis estimates the *Cervus nippon* genome size to be 2.6 G.
