# Supplementary material for: The First High-quality Reference Genome of Sika Deer Provides Insights into High-tannin Adaptation
Source: Genomics Proteomics Bioinformatics. 2022 Jun 16;21(1):203–15. doi: 10.1016/j.gpb.2022.05.008 (PMC10372904; doi:10.1016/j.gpb.2022.05.008)
Supplement: Supplementary Table S3 [file mmc20.docx]

**Table S3** **Summary of the sika deer genome assembly**

|  | **PacBio** | **PacBio + Hi-C** |
| --- | --- | --- |
| Total sequence length | 2,500,501,634 | 2,500,646,934 |
| Total sequence number | 2040 | 588 |
| Max sequence length | 93,588,229 | 143,481,735 |
| Average sequence length | 1,225,736 | 4,252,801 |
| N50 | 23,559,432 | 78,786,809 |
| N90 | 2,960,739 | 49,061,596 |

*Note*: Hi-C, high-throughput chromosome conformation capture.
