# Supplementary material for: The First High-quality Reference Genome of Sika Deer Provides Insights into High-tannin Adaptation
Source: Genomics Proteomics Bioinformatics. 2022 Jun 16;21(1):203–15. doi: 10.1016/j.gpb.2022.05.008 (PMC10372904; doi:10.1016/j.gpb.2022.05.008)
Supplement: Supplementary Table S4 [file mmc21.docx]

**Table S4** **Summary of the Hi-C assembly of chromosome length in sika deer**

| **Chromosome** | **Length (bp)** |
| --- | --- |
| chrX | 140,737,465 |
| chr1 | 143,481,735 |
| chr2 | 130,042,918 |
| chr3 | 114,820,201 |
| chr4 | 113,852,600 |
| chr5 | 105,445,150 |
| chr6 | 102,406,750 |
| chr7 | 96,153,163 |
| chr8 | 94,958,114 |
| chr9 | 90,056,637 |
| chr10 | 86,294,865 |
| chr11 | 78,786,809 |
| chr12 | 76,402,347 |
| chr13 | 75,945,954 |
| chr14 | 75,108,353 |
| chr15 | 67,521,484 |
| chr16 | 66,389,940 |
| chr17 | 62,175,516 |
| chr18 | 61,139,481 |
| chr19 | 59,074,219 |
| chr20 | 58,577,949 |
| chr21 | 57,410,528 |
| chr22 | 54,302,469 |
| chr23 | 53,848,698 |
| chr24 | 52,120,049 |
| chr25 | 51,225,997 |
| chr26 | 50,874,937 |
| chr27 | 49,061,596 |
| chr28 | 47,929,526 |
| chr29 | 43,669,067 |
| chr30 | 43,252,544 |
| chr31 | 40,873,834 |
| chr32 | 37,822,908 |

*Note*: chr, chromosome.
