# Supplementary material for: The First High-quality Reference Genome of Sika Deer Provides Insights into High-tannin Adaptation
Source: Genomics Proteomics Bioinformatics. 2022 Jun 16;21(1):203–15. doi: 10.1016/j.gpb.2022.05.008 (PMC10372904; doi:10.1016/j.gpb.2022.05.008)
Supplement: Supplementary Table S5 [file mmc22.docx]

**Table S5**  **Summary of the Cervidae genome assembly**

| **Species** | **Common name** | **Genome size (bp)** | **Ungapped length (bp)** | **Anchored ungapped length (bp)** | **Anchored rate** | **Contig N50 (bp)** | **Scaffold N50 (bp)** |
| --- | --- | --- | --- | --- | --- | --- | --- |
| *Cervus nippon* | Sika deer | 2,500,646,934 | 2,500,501,634 | 2,481,618,503 | 99.24% | 23,559,432 | 78,786,809 |
| *Cervus elaphus* | Red deer | 3,438,623,608 | 1,960,832,178 | 1,928,180,013 | 98.33% | 7944 | 107,358,006 |
| *Axis porcinus* | Hog deer | 2,676,163,295 | 2,635,847,001 | NA |  | 172,761 | 20,764,858 |
| *Hydropotes intermis* | Chinese water deer | 2,530,176,423 | 2,484,708,650 | NA |  | 131,446 | 13,818,975 |
| *Moschus moschiferus* | Siberian musk deer | 3,069,608,437 | 2,964,378,037 | NA |  | 34,785 | 11,728,851 |
| *Przewalskium albirostris* | White-lipped deer | 2,692,225,130 | 2,642,137,551 | NA |  | 39,627 | 3,769,372 |
| *Elaphurus davidianus* | Milu | 2,584,693,296 | 2,519,836,415 | NA |  | 59,950 | 2,844,142 |
| *Moschus berezovskii* | Forest musk deer | 2,818,010,997 | 2,669,554,835 | NA |  | 57,706 | 2,509,225 |
| *Rangifer tarandus* | Reindeer | 2,897,299,934 | 2,896,823,034 | NA |  | 77,671 | 1,360,739 |
| *Muntiacus crinifrons* | Black muntjac | 2,681,196,170 | 2,675,889,158 | NA |  | 8265 | 1,305,444 |
| *Muntiacus muntjak* | Indian muntjac | 2,703,710,594 | 2,607,294,293 | NA |  | 23,470 | 1,258,210 |
| *Muntiacus reevesi* | Chinese muntjac | 2,601,888,952 | 2,564,577,671 | NA |  | 72,382 | 1,221,377 |
| *Rangifer tarandus* | Reindeer | 2,832,785,815 |  | NA |  | 91,805 | 1,059,113 |
| *Odocoileus virginianus* | White-tailed Deer | 2,380,505,687 | 2,359,014,916 | NA |  | 122,019 | 850,721 |
| *Odocoileus hemionus* | Mule deer | 2,343,701,333 | 2,342,668,817 | NA |  | 113,295 | 838,758 |
| *Tragulus javanicus* | Lesser mouse-deer | 3,054,942,239 |  | NA |  | 6286 | 243,250 |
| *Moschus berezovskii* | Forest musk deer | 3,835,628,252 | 3,408,868,026 | NA |  | 24,701 | 213,462 |
| *Moschus chrysogaster* | alpine musk deer | 4,972,482,505 | 2,241,238,515 | NA |  | 3769 | 100,428 |
| *Capreolus* | Western roe deer | 2,785,377,831 | 2,741,850,513 | NA |  | 4167 | 10,458 |
| *Bos taurus* | Cattle | 2,715,853,792 | 2,715,825,630 | 2,628,394,923 | 96.78% | 25,896,116 | 103,308,737 |
