# Supplementary material for: The First High-quality Reference Genome of Sika Deer Provides Insights into High-tannin Adaptation
Source: Genomics Proteomics Bioinformatics. 2022 Jun 16;21(1):203–15. doi: 10.1016/j.gpb.2022.05.008 (PMC10372904; doi:10.1016/j.gpb.2022.05.008)
Supplement: Supplementary Table S6 [file mmc23.docx]

**Table S6**  **Assessment of the completeness and accuracy of the sika deer genome**

| **Method** | **Parameter** | **Sika deer** |
| --- | --- | --- |
| Illumina Reads Mapping and Call SNP^1^ | Coverage | 99.29% |
|  | Mapping rate | 99.36% |
|  | Ratio of heterozygous SNP | 0.38% |
|  | Ratio of homozygous SNP^2^ | 0.0011% |
| EST mapping | Total number | 2715 |
|  | Covered by genome assembly (90%)^3^ | 95.95% |
| RNA reads mapping | Total reads number (M) | 1,172.55 |
|  | Mapping rate | 93.43% |
| BUSCO | Complete BUSCOs (C) | 94.60% |
|  | Complete and single-copy BUSCOs (S) | 92.90% |
|  | Complete and duplicated BUSCOs (D) | 1.70% |
|  | Fragmented BUSCOs (F) | 2.50% |
|  | Missing BUSCOs (M) | 2.90% |
| CEGMA | Complete | 97.18% |
|  | Partial | 99.19% |

*Note*: ^1^ Using bwa, Illumina reads were mapped to the reference genome, and SAMTools was used to call high-quality SNPs. ^2^ Predicted error ratio. ^3^ Percentage of ESTs mapped to sika deer with coverage > 90%.
