# Supplementary material for: The First High-quality Reference Genome of Sika Deer Provides Insights into High-tannin Adaptation
Source: Genomics Proteomics Bioinformatics. 2022 Jun 16;21(1):203–15. doi: 10.1016/j.gpb.2022.05.008 (PMC10372904; doi:10.1016/j.gpb.2022.05.008)
Supplement: Supplementary Table S7 [file mmc24.docx]

**Table S7**  **Summary of the repeat content in the sika deer genome**

|  | **Repbase TEs** | | **RepeatModeler** | | **Combined TEs** | |
| --- | --- | --- | --- | --- | --- | --- |
|  | **Length (bp)** | **%** | **Length (bp)** | **%** | **Length (bp)** | **%** |
| DNA | 58,615,759 | 2.36 | 38,091,565 | 1.53 | 67,122,914 | 2.70 |
| LINE | 627,094,580 | 25.26 | 639,177,085 | 25.75 | 733,702,695 | 29.56 |
| LTR | 117,747,150 | 4.74 | 95,014,939 | 3.83 | 133,587,659 | 5.38 |
| SINE | 259,074,398 | 10.44 | 131,258,637 | 5.29 | 189,393,934 | 7.63 |
| Unknown | 918,332 | 0.04 | 2,436,293 | 0.10 | 2,650,074 | 0.11 |
| Total^1^ | 1,063,450,219 | 42.84 | 905,978,519 | 36.50 | 1,126,457,276 | 45.38 |
| Other^2^ | 69,041,172 | 2.78 | 25,717,291 | 1.04 | 26,376,659 | 1.06 |

*Note*: ^1^ Total: total interspersed repeats. ^2^ Other: small RNA, satellites, simple repeats, and low complexity.
