# Supplementary material for: The First High-quality Reference Genome of Sika Deer Provides Insights into High-tannin Adaptation
Source: Genomics Proteomics Bioinformatics. 2022 Jun 16;21(1):203–15. doi: 10.1016/j.gpb.2022.05.008 (PMC10372904; doi:10.1016/j.gpb.2022.05.008)
Supplement: Supplementary Table S8 [file mmc25.docx]

**Table S8**  **Comparison of the identified transposable elements among different mammalian species**

| **Species** | **Common name** | **LINEs** | **SINEs** | **LTR** | **DNA** | **Unclassified** | **Total^1^** |
| --- | --- | --- | --- | --- | --- | --- | --- |
| *Cervus nippon* | Sika deer | 29.56 | 7.63 | 5.38 | 2.70 | 0.11 | 45.38 |
| *Cervus elaphus* | Red deer | 11.64 | 6.18 | 2.86 | 1.45 | 0.02 | 22.15 |
| *Elaphurus davidianus* | Milu | 27.05 | 9.52 | 5.19 | 2.53 | 4.34 | 41.04 |
| *Rangifer tarandus* | Rein deer | 28.63 | 6.79 | 5.26 | 2.25 | 5.63 | 39.18 |
| *Capra hircus* | Goat | 26.85 | 12.35 | 4.90 | 2.61 | NA^2^ | 46.71 |
| *Ovis aries* | Sheep | 27.83 | 6.78 | 4.75 | 2.28 | NA | 42.67 |
| *Bubalus bubalis* | Water buffalo | 39.51 | 7.59 | 13.54 | 1.45 | 0.00 | 45.33 |
| *Bos taurus* | Cattle | 23.29 | 17.66 | 3.62 | 1.96 | NA | 46.54 |
| *Moschus berezovskii* | Musk deer | 23.62 | 11.35 | 4.70 | 2.34 | NA | 42.05 |
| *Giraffa camelopardalis* | Giraffe | 24.00 | 9.44 | 4.92 | 2.49 | 6.77 | 39.80 |
| *Mus musculus* | Mouse | 19.20 | 8.22 | 9.87 | 0.88 | 0 | 38.55 |
| *Homo sapiens* | Human | 20.42 | 13.14 | 8.29 | 2.84 | 0 | 44.83 |

*Note*: ^1^ Total: total interspersed repeats. ^2^ NA: repeats not available.
