# Supplementary material for: The First High-quality Reference Genome of Sika Deer Provides Insights into High-tannin Adaptation
Source: Genomics Proteomics Bioinformatics. 2022 Jun 16;21(1):203–15. doi: 10.1016/j.gpb.2022.05.008 (PMC10372904; doi:10.1016/j.gpb.2022.05.008)
Supplement: Supplementary Table S9 [file mmc26.docx]

**Table S9**  **Functional annotation of sika deer genes**

| **Category** | **Gene number** | **Ratio** |
| --- | --- | --- |
| GO | 14,161 | 66.0% |
| Interpro | 17,889 | 83.4% |
| KEGG | 19,208 | 89.6% |
| Swiss-Prot | 18,964 | 88.4% |
| TrEMBL | 19,246 | 89.7% |
| Total | 19,316 | 90.1% |
| Unannotated | 2133 | 9.9% |
