# Supplementary material for: The First High-quality Reference Genome of Sika Deer Provides Insights into High-tannin Adaptation
Source: Genomics Proteomics Bioinformatics. 2022 Jun 16;21(1):203–15. doi: 10.1016/j.gpb.2022.05.008 (PMC10372904; doi:10.1016/j.gpb.2022.05.008)
Supplement: Supplementary Table S10 [file mmc27.docx]

**Table S10**  **Summary of predicted protein-coding genes and gene characteristics**

| **Species** | **Gene number** | **Total CDS length (Mb)** | **Average CDS length** | **Exon number**  **per gene** | **Average exon length** |
| --- | --- | --- | --- | --- | --- |
| Sika deer | 21,449 | 34.69 | 1617 | 9.29 | 174 |
| Cattle | 19,994 | 32.18 | 1609 | 9.64 | 167 |
| Sheep | 20,921 | 36.44 | 1597 | 9.91 | 161 |
| Human | 20,251 | 28.54 | 1409 | 8.1 | 174 |
| Mouse | 22,159 | 32.86 | 1483 | 8.39 | 177 |
| Horse | 20,449 | 31.11 | 1521 | 9.22 | 165 |

*Note*: CDS, coding sequence.
