# Supplementary material for: The First High-quality Reference Genome of Sika Deer Provides Insights into High-tannin Adaptation
Source: Genomics Proteomics Bioinformatics. 2022 Jun 16;21(1):203–15. doi: 10.1016/j.gpb.2022.05.008 (PMC10372904; doi:10.1016/j.gpb.2022.05.008)
Supplement: Supplementary Table S11 [file mmc28.docx]

**Table S11**  **BUSCO of annotation and assembly**

|  | Complete (C) | Single copy (S) | Duplicated (D) | Fragmented (F) | Missing (M) |
| --- | --- | --- | --- | --- | --- |
| Annotation | 3907(95.2%) | 3850(93.8%) | 57(1.4%) | 101(2.5%) | 96(2.3%) |
| Assembly | 3879(94.6%) | 3810(92.9%) | 69(1.7%) | 100(2.5%) | 125(2.9%) |

*Note*: BUSCO, Benchmarking Universal Single-Copy Orthologs.
