# Supplementary material for: The First High-quality Reference Genome of Sika Deer Provides Insights into High-tannin Adaptation
Source: Genomics Proteomics Bioinformatics. 2022 Jun 16;21(1):203–15. doi: 10.1016/j.gpb.2022.05.008 (PMC10372904; doi:10.1016/j.gpb.2022.05.008)
Supplement: Supplementary Table S12 [file mmc29.docx]

**Table S12**  **Statistics for the gene families**

| **Species** | **Common name** | **Single-copy orthologs** | **Unique orthologs** | **Multiple-copy orthologs** | **Other** |
| --- | --- | --- | --- | --- | --- |
| *Homo Sapiens* | Human | 5265 | 22 | 16,523 | 678 |
| *Mus musculus* | Mouse | 5390 | 18 | 15,743 | 792 |
| *Balaenoptera acutorostrata* | Minke whale | 5303 | 19 | 12,275 | 1010 |
| *Camelus bactrianus* | Bactrian camel | 5392 | 18 | 12,102 | 1201 |
| *Camelus dromedarius* | Dromedary | 5255 | 0 | 11,858 | 1397 |
| *Sus scrofa* | Pig | 4432 | 0 | 16,306 | 1266 |
| *Giraffa camelopardalis* | Giraffe | 5368 | 1 | 11,472 | 1255 |
| *Okapia johnstoni* | Okapi | 5363 | 0 | 11,394 | 1281 |
| *Moschus moschiferus* | Musk deer | 4744 | 27 | 15,969 | 1547 |
| *Bos taurus* | Cattle | 5386 | 65 | 13,864 | 913 |
| *Bos grunniens* | Yak | 5342 | 51 | 14,227 | 857 |
| *Ovris aries* | Sheep | 5276 | 58 | 14,402 | 889 |
| *Capra hircus* | Goat | 5416 | 0 | 14,382 | 825 |
| *Odocoileus virginianus* | White-tailed deer | 5251 | 1 | 14,745 | 800 |
| *Elaphurus davidianus* | Milu | 5300 | 36 | 12,748 | 1133 |
| *Cervus elaphus* | Red deer | 4308 | 1 | 12,944 | 1937 |
| *Rangifer tarandus* | Reindeer | 4186 | 2 | 13,575 | 2034 |
| *Hydropotes inermis* | Roe deer | 5429 | 0 | 10,991 | 1260 |
| *Cervus nippon* | Sika deer | 4918 | 0 | 13,486 | 1284 |
