# Supplementary material for: The First High-quality Reference Genome of Sika Deer Provides Insights into High-tannin Adaptation
Source: Genomics Proteomics Bioinformatics. 2022 Jun 16;21(1):203–15. doi: 10.1016/j.gpb.2022.05.008 (PMC10372904; doi:10.1016/j.gpb.2022.05.008)
Supplement: Supplementary Table S13 [file mmc30.docx]

**Table S13**  **Positively selected genes identified in sika deer**

| **Symbol** | **Name** | ***P* value** |
| --- | --- | --- |
| CDC23 | Cell division cycle protein 23 homolog | 0 |
| BAD | Bcl2-associated agonist of cell death | 0 |
| POLR3H | DNA-directed RNA polymerase III subunit RPC8 | 3E-09 |
| HSPA13 | Heat shock 70 kDa protein 13 | 2.3E-08 |
| DNAJC11 | DnaJ homolog subfamily C member 11 | 0 |
| USHBP1 | Usher syndrome type-1C protein-binding protein 1 | 0 |
| ACAA2 | 3-ketoacyl-CoA thiolase, mitochondrial | 0 |
| TBC1D13 | TBC1 domain family member 13 | 0 |
| JMJD4 | JmjC domain-containing protein 4 | 0 |
| ZW10 | Centromere/kinetochore protein zw10 homolog | 0 |
| EDF1 | Endothelial differentiation-related factor 1 | 0 |
| DDX42 | ATP-dependent RNA helicase DDX42 | 6.1E-08 |
| AGA | N(4)-(beta-N-acetylglucosaminyl)-L-asparaginase | 0 |
| GPN2 | GPN-loop GTPase 2 | 2E-09 |
| SLC25A19 | Mitochondrial thiamine pyrophosphate carrier | 1.45E-03 |
| UFC1 | Ubiquitin-fold modifier-conjugating enzyme 1 | 1.05E-07 |
| PEX12 | Peroxisome assembly protein 12 | 0 |
| ZC3HC1 | Nuclear-interacting partner of ALK | 0 |
| TGM1 | Protein-glutamine gamma-glutamyltransferase K | 0 |
| Med19 | Mediator of RNA polymerase II transcription subunit 19 | 0 |
| NPAS4 | Neuronal PAS domain-containing protein 4 | 0 |
| TRMT10C | tRNA methyltransferase 10 homolog C | 0 |
| RTF1 | RNA polymerase-associated protein RTF1 homolog | 1.21E-06 |
| RCHY1 | RING finger and CHY zinc finger domain-containing protein 1 | 0 |
| UBLCP1 | Ubiquitin-like domain-containing CTD phosphatase 1 | 0 |
| TOLLIP | Toll-interacting protein | 0 |
| MRPL44 | 39S ribosomal protein L44, mitochondrial | 0 |
| MED1 | Mediator of RNA polymerase II transcription subunit 1 | 0 |
| MORN5 | MORN repeat-containing protein 5 | 0 |
| ZMYND12 | Zinc finger MYND domain-containing protein 12 | 1.51E-03 |
| Pkdcc | Extracellular tyrosine-protein kinase PKDCC | 0 |
| TMF1 | TATA element modulatory factor | 0 |
| PPP1R36 | Protein phosphatase 1 regulatory subunit 36 | 0 |
| PRKCSH | Glucosidase 2 subunit beta | 2.24E-05 |
| MLH3 | DNA mismatch repair protein Mlh3 | 0 |
| - | UPF0449 protein C19orf25 homolog | 5.14E-06 |
| INHA | Inhibin alpha chain | 0 |
| CMBL | Carboxymethylenebutenolidase homolog | 7.85E-04 |
| TTC22 | Tetratricopeptide repeat protein 22 | 3.44E-05 |
| NKAPD1 | Uncharacterized protein NKAPD1 | 0 |
| DNAJB5 | DnaJ homolog subfamily B member 5 | 0 |
| TBATA | Protein TBATA | 0 |
| RCSD1 | CapZ-interacting protein | 0 |
| CD34 | Hematopoietic progenitor cell antigen CD34 | 2.29E-05 |
| KCNE2 | Potassium voltage-gated channel subfamily E member 2 | 5E-09 |
| ACRBP | Acrosin-binding protein (Fragment) | 0 |
| CD72 | B-cell differentiation antigen CD72 | 9.1E-08 |
| DDN | Dendrin | 0 |
| CCDC155 | Protein KASH5 | 0 |
| Slfnl1 | Schlafen-like protein 1 | 0 |
| TMEM139 | Transmembrane protein 139 | 0 |
| IZUMO3 | Izumo sperm-egg fusion protein 3 | 0 |
| HINFP | Histone H4 transcription factor | 0 |
| DHX30 | Putative ATP-dependent RNA helicase DHX30 | 0 |
| GRAMD1C | GRAM domain-containing protein 1C | 0 |
