# Supplementary material for: The First High-quality Reference Genome of Sika Deer Provides Insights into High-tannin Adaptation
Source: Genomics Proteomics Bioinformatics. 2022 Jun 16;21(1):203–15. doi: 10.1016/j.gpb.2022.05.008 (PMC10372904; doi:10.1016/j.gpb.2022.05.008)
Supplement: Supplementary Table S15 [file mmc32.docx]

**Table S15**  **Functionally enriched KEGG pathway categories of sika deer expanded genes**

| **ID** | **Categories** | ***P* value** | ***P* adj** |
| --- | --- | --- | --- |
| ko04740 | Olfactory transduction | 0 | 0 |
| ko03010 | Ribosome | 1.13E-32 | 1.31E-30 |
| ko03011 | Ribosome | 1.13E-32 | 1.31E-30 |
| ko04147 | Exosome | 6.18E-13 | 5.39E-11 |
| ko03000 | Transcription factors | 8.48E-13 | 5.92E-11 |
| ko05034 | Alcoholism | 1.07E-12 | 6.24E-11 |
| ko04812 | Cytoskeleton proteins | 2.41E-12 | 1.20E-10 |
| ko03036 | Chromosome and associated proteins | 5.62E-12 | 2.45E-10 |
| ko04030 | G protein-coupled receptors | 6.66E-12 | 2.58E-10 |
| ko05322 | Systemic lupus erythematosus | 4.86E-11 | 1.70E-09 |
| ko04031 | GTP-binding proteins | 1.23E-09 | 3.89E-08 |
| ko00140 | Steroid hormone biosynthesis | 5.21E-08 | 1.51E-06 |
| ko00310 | Lysine degradation | 6.00E-08 | 1.61E-06 |
| ko04390 | Hippo signaling pathway | 1.05E-07 | 2.61E-06 |
| ko04612 | Antigen processing and presentation | 1.54E-07 | 3.59E-06 |
| ko04626 | Plant-pathogen interaction | 1.78E-07 | 3.87E-06 |
| ko00830 | Retinol metabolism | 2.10E-07 | 4.32E-06 |
| ko05150 | Staphylococcus aureus infection | 6.99E-07 | 1.35E-05 |
| ko00982 | Drug metabolism - cytochrome P450 | 4.17E-06 | 7.65E-05 |
| ko00053 | Ascorbate and aldarate metabolism | 5.24E-06 | 9.14E-05 |
| ko05310 | Asthma | 6.50E-06 | 1.08E-04 |
| ko04145 | Phagosome | 9.35E-06 | 1.48E-04 |
| ko00040 | Pentose and glucuronate interconversions | 1.35E-05 | 2.05E-04 |
| ko01009 | Protein phosphatase and associated proteins | 1.99E-05 | 2.90E-04 |
| ko05130 | Pathogenic Escherichia coli infection | 4.98E-05 | 6.96E-04 |
| ko03019 | Messenger RNA Biogenesis | 5.43E-05 | 7.29E-04 |
| ko00860 | Porphyrin and chlorophyll metabolism | 1.15E-04 | 1.48E-03 |
| ko03051 | Proteasome | 1.18E-04 | 1.48E-03 |
| ko04350 | TGF-beta signaling pathway | 1.29E-04 | 1.56E-03 |
| ko04672 | Intestinal immune network for IgA production | 1.75E-04 | 2.04E-03 |
| ko05031 | Amphetamine addiction | 2.03E-04 | 2.29E-03 |
| ko04744 | Phototransduction | 2.26E-04 | 2.46E-03 |
| ko04540 | Gap junction | 2.39E-04 | 2.53E-03 |
| ko05332 | Graft-versus-host disease | 2.68E-04 | 2.76E-03 |
| ko04110 | Cell cycle | 2.92E-04 | 2.90E-03 |
| ko04391 | Hippo signaling pathway - fly | 3.00E-04 | 2.90E-03 |
| ko05323 | Rheumatoid arthritis | 3.07E-04 | 2.90E-03 |
| ko00980 | Metabolism of xenobiotics by cytochrome P450 | 3.40E-04 | 3.13E-03 |
| ko04940 | Type I diabetes mellitus | 3.91E-04 | 3.50E-03 |
| ko05416 | Viral myocarditis | 4.40E-04 | 3.84E-03 |
| ko00534 | Glycosaminoglycan biosynthesis - heparan sulfate/heparin | 5.16E-04 | 4.39E-03 |
| ko00512 | Mucin type O-glycan biosynthesis | 5.98E-04 | 4.86E-03 |
| ko05033 | Nicotine addiction | 5.98E-04 | 4.86E-03 |
| ko04114 | Oocyte meiosis | 7.09E-04 | 5.50E-03 |
| ko05030 | Cocaine addiction | 6.95E-04 | 5.50E-03 |
| ko00983 | Drug metabolism - other enzymes | 9.07E-04 | 6.88E-03 |
| ko01020 | Enzyme-linked receptors | 9.34E-04 | 6.93E-03 |
| ko05012 | Parkinson's disease | 9.77E-04 | 6.96E-03 |
| ko05204 | Chemical carcinogenesis | 9.69E-04 | 6.96E-03 |
| ko05010 | Alzheimer's disease | 1.00E-03 | 7.01E-03 |
| ko00604 | Glycosphingolipid biosynthesis - ganglio series | 1.18E-03 | 8.11E-03 |
| ko00190 | Oxidative phosphorylation | 1.30E-03 | 8.47E-03 |
| ko04218 | Cellular senescence | 1.27E-03 | 8.47E-03 |
| ko05330 | Allograft rejection | 1.31E-03 | 8.47E-03 |
| ko05016 | Huntington's disease | 1.40E-03 | 8.87E-03 |
| ko05166 | HTLV-I infection | 1.45E-03 | 9.04E-03 |
| ko04016 | MAPK signaling pathway - plant | 1.49E-03 | 9.09E-03 |
| ko05320 | Autoimmune thyroid disease | 2.39E-03 | 1.44E-02 |
| ko04730 | Long-term depression | 2.88E-03 | 1.71E-02 |
| ko00910 | Nitrogen metabolism | 3.06E-03 | 1.78E-02 |
| ko04728 | Dopaminergic synapse | 4.09E-03 | 2.34E-02 |
| ko04015 | Rap1 signaling pathway | 4.38E-03 | 2.46E-02 |
| ko04261 | Adrenergic signaling in cardiomyocytes | 5.24E-03 | 2.90E-02 |
| ko03009 | Ribosome biogenesis | 5.33E-03 | 2.91E-02 |
| ko01003 | Glycosyltransferases | 5.52E-03 | 2.97E-02 |
| ko03041 | Spliceosome | 5.65E-03 | 2.99E-02 |
| ko04516 | Cell adhesion molecules and their ligands | 6.01E-03 | 3.13E-02 |
| ko05321 | Inflammatory bowel disease (IBD) | 6.40E-03 | 3.28E-02 |
| ko04745 | Phototransduction – fly | 9.16E-03 | 4.63E-02 |
