# Supplementary material for: The First High-quality Reference Genome of Sika Deer Provides Insights into High-tannin Adaptation
Source: Genomics Proteomics Bioinformatics. 2022 Jun 16;21(1):203–15. doi: 10.1016/j.gpb.2022.05.008 (PMC10372904; doi:10.1016/j.gpb.2022.05.008)
Supplement: Supplementary Table S16 [file mmc33.docx]

**Table S16**  **Functionally enriched GO categories of sika deer expanded genes**

| **ID** | **Categories** | ***P* value** | ***P* adj** |
| --- | --- | --- | --- |
| GO:0003676 | nucleic acid binding | 0 | 0 |
| GO:0004984 | olfactory receptor activity | 0 | 0 |
| GO:0006355 | regulation of transcription, DNA-templated | 0 | 0 |
| GO:0007186 | G-protein coupled receptor signaling pathway | 0 | 0 |
| GO:0046872 | metal ion binding | 0 | 0 |
| GO:0005840 | ribosome | 7.00E-50 | 7.13E-48 |
| GO:0003735 | structural constituent of ribosome | 8.70E-47 | 7.59E-45 |
| GO:0006412 | translation | 1.56E-44 | 1.19E-42 |
| GO:0005882 | intermediate filament | 7.16E-35 | 4.86E-33 |
| GO:0019068 | virion assembly | 1.13E-31 | 6.92E-30 |
| GO:0005198 | structural molecule activity | 9.70E-31 | 5.39E-29 |
| GO:0016887 | ATPase activity | 1.65E-22 | 8.38E-21 |
| GO:0016032 | viral process | 5.34E-22 | 2.51E-20 |
| GO:0045095 | keratin filament | 1.56E-21 | 6.81E-20 |
| GO:0008076 | voltage-gated potassium channel complex | 1.04E-19 | 4.25E-18 |
| GO:0042626 | ATPase activity, coupled to transmembrane movement of substances | 2.73E-19 | 1.04E-17 |
| GO:0019028 | viral capsid | 7.47E-13 | 2.68E-11 |
| GO:0005249 | voltage-gated potassium channel activity | 9.79E-13 | 3.32E-11 |
| GO:0005622 | intracellular | 2.75E-12 | 8.84E-11 |
| GO:0003700 | transcription factor activity, sequence-specific DNA binding | 3.03E-12 | 9.25E-11 |
| GO:0004930 | G-protein coupled receptor activity | 5.08E-12 | 1.48E-10 |
| GO:0007156 | homophilic cell adhesion via plasma membrane adhesion molecules | 4.89E-11 | 1.36E-09 |
| GO:0000786 | nucleosome | 1.96E-10 | 5.22E-09 |
| GO:0004890 | GABA-A receptor activity | 3.34E-10 | 8.49E-09 |
| GO:0042613 | MHC class II protein complex | 5.78E-10 | 1.41E-08 |
| GO:0016021 | integral component of membrane | 1.45E-09 | 3.41E-08 |
| GO:0043565 | sequence-specific DNA binding | 3.88E-09 | 8.78E-08 |
| GO:0004888 | transmembrane signaling receptor activity | 4.05E-09 | 8.85E-08 |
| GO:0005887 | integral component of plasma membrane | 5.79E-09 | 1.22E-07 |
| GO:0016020 | membrane | 8.43E-09 | 1.72E-07 |
| GO:0016712 | oxidoreductase activity, acting on paired donors, with incorporation or reduction of molecular oxygen, reduced flavin or flavoprotein as one donor, and incorporation of one atom of oxygen | 9.81E-09 | 1.93E-07 |
| GO:0007166 | cell surface receptor signaling pathway | 2.26E-08 | 4.32E-07 |
| GO:0005886 | plasma membrane | 2.57E-08 | 4.77E-07 |
| GO:0004523 | RNA-DNA hybrid ribonuclease activity | 4.00E-08 | 7.18E-07 |
| GO:0006810 | transport | 4.34E-08 | 7.57E-07 |
| GO:0019904 | protein domain specific binding | 4.69E-08 | 7.96E-07 |
| GO:0051260 | protein homooligomerization | 5.21E-08 | 8.60E-07 |
| GO:0006813 | potassium ion transport | 8.70E-08 | 1.40E-06 |
| GO:0019001 | guanyl nucleotide binding | 1.78E-07 | 2.71E-06 |
| GO:0031683 | G-protein beta/gamma-subunit complex binding | 1.78E-07 | 2.71E-06 |
| GO:0016758 | transferase activity, transferring hexosyl groups | 2.03E-07 | 3.03E-06 |
| GO:0019882 | antigen processing and presentation | 3.74E-07 | 5.43E-06 |
| GO:0004012 | phospholipid-translocating ATPase activity | 6.88E-07 | 9.55E-06 |
| GO:0015914 | phospholipid transport | 6.88E-07 | 9.55E-06 |
| GO:0004983 | neuropeptide Y receptor activity | 1.08E-06 | 1.44E-05 |
| GO:0008021 | synaptic vesicle | 1.08E-06 | 1.44E-05 |
| GO:0046982 | protein heterodimerization activity | 1.16E-06 | 1.51E-05 |
| GO:0005003 | ephrin receptor activity | 2.56E-06 | 3.26E-05 |
| GO:0005525 | GTP binding | 2.65E-06 | 3.31E-05 |
| GO:0007264 | small GTPase-mediated signal transduction | 4.37E-06 | 5.35E-05 |
| GO:0007214 | gamma-aminobutyric acid signaling pathway | 4.99E-06 | 5.98E-05 |
| GO:0048013 | ephrin receptor signaling pathway | 9.58E-06 | 1.13E-04 |
| GO:0005230 | extracellular ligand-gated ion channel activity | 1.30E-05 | 1.50E-04 |
| GO:0051082 | unfolded protein binding | 1.54E-05 | 1.75E-04 |
| GO:0004180 | carboxypeptidase activity | 2.30E-05 | 2.47E-04 |
| GO:0004459 | L-lactate dehydrogenase activity | 2.30E-05 | 2.47E-04 |
| GO:0005044 | scavenger receptor activity | 2.32E-05 | 2.47E-04 |
| GO:0006811 | ion transport | 2.34E-05 | 2.47E-04 |
| GO:0000166 | nucleotide binding | 2.65E-05 | 2.75E-04 |
| GO:0003924 | GTPase activity | 3.67E-05 | 3.68E-04 |
| GO:0007188 | adenylate cyclase-modulating G-protein coupled receptor signaling pathway | 3.63E-05 | 3.68E-04 |
| GO:0004114 | 3',5'-cyclic-nucleotide phosphodiesterase activity | 3.89E-05 | 3.72E-04 |
| GO:0004143 | diacylglycerol kinase activity | 3.86E-05 | 3.72E-04 |
| GO:0007205 | protein kinase C-activating G-protein coupled receptor signaling pathway | 3.86E-05 | 3.72E-04 |
| GO:0005667 | transcription factor complex | 5.64E-05 | 5.15E-04 |
| GO:0007155 | cell adhesion | 5.65E-05 | 5.15E-04 |
| GO:0050661 | NADP binding | 5.64E-05 | 5.15E-04 |
| GO:0008009 | chemokine activity | 6.60E-05 | 5.93E-04 |
| GO:0005874 | microtubule | 7.30E-05 | 6.37E-04 |
| GO:0007017 | microtubule-based process | 7.30E-05 | 6.37E-04 |
| GO:0007169 | transmembrane receptor protein tyrosine kinase signaling pathway | 7.60E-05 | 6.54E-04 |
| GO:0003956 | NAD(P)+-protein-arginine ADP-ribosyltransferase activity | 1.06E-04 | 8.39E-04 |
| GO:0004952 | dopamine neurotransmitter receptor activity | 1.06E-04 | 8.39E-04 |
| GO:0004977 | melanocortin receptor activity | 1.06E-04 | 8.39E-04 |
| GO:0005549 | odorant binding | 1.06E-04 | 8.39E-04 |
| GO:0008097 | 5S rRNA binding | 1.06E-04 | 8.39E-04 |
| GO:0016907 | G-protein coupled acetylcholine receptor activity | 1.06E-04 | 8.39E-04 |
| GO:0000785 | chromatin | 1.49E-04 | 1.15E-03 |
| GO:0031492 | nucleosomal DNA binding | 1.49E-04 | 1.15E-03 |
| GO:0004181 | metallocarboxypeptidase activity | 1.60E-04 | 1.22E-03 |
| GO:0019058 | viral life cycle | 2.26E-04 | 1.70E-03 |
| GO:0016705 | oxidoreductase activity, acting on paired donors, with incorporation or reduction of molecular oxygen | 2.39E-04 | 1.78E-03 |
| GO:0008146 | sulfotransferase activity | 2.42E-04 | 1.78E-03 |
| GO:0006935 | chemotaxis | 2.61E-04 | 1.90E-03 |
| GO:0009607 | response to biotic stimulus | 3.26E-04 | 2.34E-03 |
| GO:0005581 | collagen trimer | 4.86E-04 | 3.38E-03 |
| GO:0006913 | nucleocytoplasmic transport | 4.86E-04 | 3.38E-03 |
| GO:0045296 | cadherin binding | 4.86E-04 | 3.38E-03 |
| GO:0004950 | chemokine receptor activity | 5.08E-04 | 3.49E-03 |
| GO:0016337 | single organismal cell-cell adhesion | 5.43E-04 | 3.69E-03 |
| GO:0001664 | G-protein coupled receptor binding | 6.02E-04 | 3.88E-03 |
| GO:0004499 | N,N-dimethylaniline monooxygenase activity | 6.02E-04 | 3.88E-03 |
| GO:0016820 | hydrolase activity, acting on acid anhydrides, catalyzing transmembrane movement of substances | 6.02E-04 | 3.88E-03 |
| GO:0020037 | heme binding | 6.03E-04 | 3.88E-03 |
| GO:0031110 | regulation of microtubule polymerization or depolymerization | 6.02E-04 | 3.88E-03 |
| GO:0006414 | translational elongation | 7.81E-04 | 4.97E-03 |
| GO:0006457 | protein folding | 8.67E-04 | 5.46E-03 |
| GO:0015934 | large ribosomal subunit | 9.08E-04 | 5.66E-03 |
| GO:0004871 | signal transducer activity | 1.17E-03 | 7.25E-03 |
| GO:0005328 | neurotransmitter:sodium symporter activity | 1.31E-03 | 7.93E-03 |
| GO:0040007 | growth | 1.31E-03 | 7.93E-03 |
| GO:0005200 | structural constituent of cytoskeleton | 1.41E-03 | 8.43E-03 |
| GO:0006955 | immune response | 1.64E-03 | 9.74E-03 |
| GO:0004252 | serine-type endopeptidase activity | 1.80E-03 | 1.06E-02 |
| GO:0004427 | inorganic diphosphatase activity | 2.24E-03 | 1.12E-02 |
| GO:0004618 | phosphoglycerate kinase activity | 2.24E-03 | 1.12E-02 |
| GO:0004726 | non-membrane-spanning protein tyrosine phosphatase activity | 2.24E-03 | 1.12E-02 |
| GO:0004800 | thyroxine 5'-deiodinase activity | 2.24E-03 | 1.12E-02 |
| GO:0005094 | Rho GDP-dissociation inhibitor activity | 2.24E-03 | 1.12E-02 |
| GO:0005158 | insulin receptor binding | 2.21E-03 | 1.12E-02 |
| GO:0006796 | phosphate-containing compound metabolic process | 2.24E-03 | 1.12E-02 |
| GO:0007269 | neurotransmitter secretion | 2.24E-03 | 1.12E-02 |
| GO:0007420 | brain development | 2.24E-03 | 1.12E-02 |
| GO:0015016 | [heparan sulfate]-glucosamine N-sulfotransferase activity | 2.24E-03 | 1.12E-02 |
| GO:0015074 | DNA integration | 1.96E-03 | 1.12E-02 |
| GO:0016471 | vacuolar proton-transporting V-type ATPase complex | 2.24E-03 | 1.12E-02 |
| GO:0016594 | glycine binding | 2.24E-03 | 1.12E-02 |
| GO:0016934 | extracellular-glycine-gated chloride channel activity | 2.24E-03 | 1.12E-02 |
| GO:0022824 | transmitter-gated ion channel activity | 2.24E-03 | 1.12E-02 |
| GO:0032968 | positive regulation of transcription elongation from RNA polymerase II promoter | 2.24E-03 | 1.12E-02 |
| GO:0035329 | hippo signaling | 2.24E-03 | 1.12E-02 |
| GO:0045263 | proton-transporting ATP synthase complex, coupling factor F(o) | 2.24E-03 | 1.12E-02 |
| GO:0055085 | transmembrane transport | 2.44E-03 | 1.21E-02 |
| GO:0006950 | response to stress | 3.28E-03 | 1.60E-02 |
| GO:0015992 | proton transport | 3.28E-03 | 1.60E-02 |
| GO:0005216 | ion channel activity | 3.45E-03 | 1.67E-02 |
| GO:0004601 | peroxidase activity | 4.80E-03 | 2.27E-02 |
| GO:0005742 | mitochondrial outer membrane translocase complex | 4.80E-03 | 2.27E-02 |
| GO:0007179 | transforming growth factor beta receptor signaling pathway | 4.80E-03 | 2.27E-02 |
| GO:0006821 | chloride transport | 4.86E-03 | 2.29E-02 |
| GO:0006006 | glucose metabolic process | 6.86E-03 | 3.17E-02 |
| GO:0019773 | proteasome core complex, alpha-subunit complex | 6.86E-03 | 3.17E-02 |
| GO:0005578 | proteinaceous extracellular matrix | 6.94E-03 | 3.19E-02 |
| GO:0006836 | neurotransmitter transport | 7.05E-03 | 3.21E-02 |
| GO:0006413 | translational initiation | 7.62E-03 | 3.42E-02 |
| GO:0030286 | dynein complex | 7.62E-03 | 3.42E-02 |
| GO:0002224 | toll-like receptor signaling pathway | 1.03E-02 | 3.81E-02 |
| GO:0004146 | dihydrofolate reductase activity | 1.03E-02 | 3.81E-02 |
| GO:0004356 | glutamate-ammonia ligase activity | 1.03E-02 | 3.81E-02 |
| GO:0004392 | heme oxygenase (decyclizing) activity | 1.03E-02 | 3.81E-02 |
| GO:0004563 | beta-N-acetylhexosaminidase activity | 1.03E-02 | 3.81E-02 |
| GO:0004656 | procollagen-proline 4-dioxygenase activity | 1.03E-02 | 3.81E-02 |
| GO:0005030 | neurotrophin receptor activity | 1.03E-02 | 3.81E-02 |
| GO:0005853 | eukaryotic translation elongation factor 1 complex | 1.03E-02 | 3.81E-02 |
| GO:0005960 | glycine cleavage complex | 9.24E-03 | 3.81E-02 |
| GO:0006471 | protein ADP-ribosylation | 9.80E-03 | 3.81E-02 |
| GO:0006542 | glutamine biosynthetic process | 1.03E-02 | 3.81E-02 |
| GO:0006545 | glycine biosynthetic process | 1.03E-02 | 3.81E-02 |
| GO:0006788 | heme oxidation | 1.03E-02 | 3.81E-02 |
| GO:0006885 | regulation of pH | 9.80E-03 | 3.81E-02 |
| GO:0007195 | adenylate cyclase-inhibiting dopamine receptor signaling pathway | 1.03E-02 | 3.81E-02 |
| GO:0009263 | deoxyribonucleotide biosynthetic process | 1.03E-02 | 3.81E-02 |
| GO:0009408 | response to heat | 9.24E-03 | 3.81E-02 |
| GO:0015321 | sodium-dependent phosphate transmembrane transporter activity | 1.03E-02 | 3.81E-02 |
| GO:0015385 | sodium:proton antiporter activity | 9.80E-03 | 3.81E-02 |
| GO:0016494 | C-X-C chemokine receptor activity | 1.03E-02 | 3.81E-02 |
| GO:0016607 | nuclear speck | 1.03E-02 | 3.81E-02 |
| GO:0019464 | glycine decarboxylation via glycine cleavage system | 9.24E-03 | 3.81E-02 |
| GO:0019538 | protein metabolic process | 1.03E-02 | 3.81E-02 |
| GO:0031032 | actomyosin structure organization | 9.24E-03 | 3.81E-02 |
| GO:0031072 | heat shock protein binding | 9.80E-03 | 3.81E-02 |
| GO:0043560 | insulin receptor substrate binding | 1.03E-02 | 3.81E-02 |
| GO:0044341 | sodium-dependent phosphate transport | 1.03E-02 | 3.81E-02 |
| GO:0046541 | saliva secretion | 1.03E-02 | 3.81E-02 |
| GO:0070461 | SAGA-type complex | 1.03E-02 | 3.81E-02 |
| GO:0051015 | actin filament binding | 1.17E-02 | 4.31E-02 |
