# Supplementary material for: The First High-quality Reference Genome of Sika Deer Provides Insights into High-tannin Adaptation
Source: Genomics Proteomics Bioinformatics. 2022 Jun 16;21(1):203–15. doi: 10.1016/j.gpb.2022.05.008 (PMC10372904; doi:10.1016/j.gpb.2022.05.008)
Supplement: Supplementary Table S17 [file mmc34.docx]

**Table S17**  **Functionally enriched KEGG pathway categories of sika deer contracted genes**

| **ID** | **Categories** | ***P* value** | ***P* adj** |
| --- | --- | --- | --- |
| ko04030 | G protein-coupled receptors | 0 | 0 |
| ko04740 | Olfactory transduction | 0 | 0 |
| ko04040 | Ion channels | 3.24E-11 | 3.52E-09 |
| ko00590 | Arachidonic acid metabolism | 5.27E-11 | 4.29E-09 |
| ko05020 | Prion diseases | 3.15E-08 | 2.05E-06 |
| ko02000 | Transporters | 1.29E-07 | 6.99E-06 |
| ko04330 | Notch signaling pathway | 1.59E-07 | 7.43E-06 |
| ko04975 | Fat digestion and absorption | 2.20E-06 | 8.95E-05 |
| ko00591 | Linoleic acid metabolism | 3.46E-06 | 1.13E-04 |
| ko04516 | Cell adhesion molecules and their ligands | 3.16E-06 | 1.13E-04 |
| ko04020 | Calcium signaling pathway | 1.57E-05 | 4.66E-04 |
| ko00199 | Cytochrome P450 | 3.40E-05 | 7.91E-04 |
| ko04320 | Dorso-ventral axis formation | 3.36E-05 | 7.91E-04 |
| ko04960 | Aldosterone-regulated sodium reabsorption | 3.28E-05 | 7.91E-04 |
| ko04668 | TNF signaling pathway | 5.74E-05 | 1.25E-03 |
| ko00592 | alpha-Linolenic acid metabolism | 6.93E-05 | 1.34E-03 |
| ko05146 | Amoebiasis | 7.01E-05 | 1.34E-03 |
| ko03320 | PPAR signaling pathway | 7.50E-05 | 1.36E-03 |
| ko04014 | Ras signaling pathway | 1.25E-04 | 2.11E-03 |
| ko04621 | NOD-like receptor signaling pathway | 1.29E-04 | 2.11E-03 |
| ko04978 | Mineral absorption | 1.37E-04 | 2.13E-03 |
| ko04973 | Carbohydrate digestion and absorption | 1.57E-04 | 2.32E-03 |
| ko05164 | Influenza A | 1.73E-04 | 2.45E-03 |
| ko04052 | Cytokines | 2.28E-04 | 3.10E-03 |
| ko05222 | Small cell lung cancer | 2.65E-04 | 3.45E-03 |
| ko04972 | Pancreatic secretion | 2.92E-04 | 3.66E-03 |
| ko04380 | Osteoclast differentiation | 3.53E-04 | 4.26E-03 |
| ko04360 | Axon guidance | 3.78E-04 | 4.41E-03 |
| ko05134 | Legionellosis | 3.96E-04 | 4.45E-03 |
| ko04010 | MAPK signaling pathway | 4.55E-04 | 4.95E-03 |
| ko04622 | RIG-I-like receptor signaling pathway | 5.43E-04 | 5.53E-03 |
| ko04658 | Th1 and Th2 cell differentiation | 5.28E-04 | 5.53E-03 |
| ko00062 | Fatty acid elongation | 6.07E-04 | 5.82E-03 |
| ko04657 | IL-17 signaling pathway | 5.91E-04 | 5.82E-03 |
| ko00565 | Ether lipid metabolism | 6.94E-04 | 6.46E-03 |
| ko04660 | T cell receptor signaling pathway | 7.36E-04 | 6.66E-03 |
| ko04742 | Taste transduction | 8.57E-04 | 7.55E-03 |
| ko04210 | Apoptosis | 1.19E-03 | 1.02E-02 |
| ko04726 | Serotonergic synapse | 1.29E-03 | 1.08E-02 |
| ko04066 | HIF-1 signaling pathway | 1.68E-03 | 1.34E-02 |
| ko05152 | Tuberculosis | 1.68E-03 | 1.34E-02 |
| ko05167 | Kaposi's sarcoma-associated herpesvirus infection | 2.24E-03 | 1.74E-02 |
| ko04662 | B cell receptor signaling pathway | 2.69E-03 | 2.03E-02 |
| ko05165 | Human papillomavirus infection | 2.74E-03 | 2.03E-02 |
| ko04750 | Inflammatory mediator regulation of TRP channels | 3.11E-03 | 2.26E-02 |
| ko04121 | Ubiquitin system | 3.49E-03 | 2.46E-02 |
| ko04217 | Necroptosis | 3.55E-03 | 2.46E-02 |
| ko04919 | Thyroid hormone signaling pathway | 3.72E-03 | 2.50E-02 |
| ko04930 | Type II diabetes mellitus | 3.75E-03 | 2.50E-02 |
| ko01522 | Endocrine resistance | 4.86E-03 | 3.11E-02 |
| ko04620 | Toll-like receptor signaling pathway | 4.86E-03 | 3.11E-02 |
| ko05160 | Hepatitis C | 5.48E-03 | 3.37E-02 |
| ko99992 | Membrane and intracellular structural | 5.41E-03 | 3.37E-02 |
| ko04971 | Gastric acid secretion | 5.69E-03 | 3.44E-02 |
| ko05211 | Renal cell carcinoma | 7.40E-03 | 4.39E-02 |
| ko04911 | Insulin secretion | 8.33E-03 | 4.76E-02 |
| ko04925 | Aldosterone synthesis and secretion | 8.33E-03 | 4.76E-02 |
