# Supplementary material for: The First High-quality Reference Genome of Sika Deer Provides Insights into High-tannin Adaptation
Source: Genomics Proteomics Bioinformatics. 2022 Jun 16;21(1):203–15. doi: 10.1016/j.gpb.2022.05.008 (PMC10372904; doi:10.1016/j.gpb.2022.05.008)
Supplement: Supplementary Table S18 [file mmc35.docx]

**Table S18**  **Functionally enriched GO categories of sika deer contracted genes**

| **ID** | **Categories** | ***P* value** | ***P* adj** |
| --- | --- | --- | --- |
| GO:0004930 | G-protein coupled receptor activity | 0 | 0 |
| GO:0004984 | olfactory receptor activity | 0 | 0 |
| GO:0005509 | calcium ion binding | 0 | 0 |
| GO:0005515 | protein binding | 0 | 0 |
| GO:0007186 | G-protein coupled receptor signaling pathway | 0 | 0 |
| GO:0006820 | anion transport | 2.21E-12 | 1.45E-10 |
| GO:0016021 | integral component of membrane | 3.23E-12 | 1.82E-10 |
| GO:0005506 | iron ion binding | 4.97E-12 | 2.45E-10 |
| GO:0005615 | extracellular space | 3.30E-10 | 1.44E-08 |
| GO:0008272 | sulfate transport | 4.48E-10 | 1.60E-08 |
| GO:0015116 | sulfate transmembrane transporter activity | 4.48E-10 | 1.60E-08 |
| GO:0005452 | inorganic anion exchanger activity | 6.21E-09 | 2.04E-07 |
| GO:0022857 | transmembrane transporter activity | 7.14E-09 | 2.16E-07 |
| GO:0008271 | secondary active sulfate transmembrane transporter activity | 1.97E-08 | 5.54E-07 |
| GO:0005215 | transporter activity | 2.88E-08 | 7.56E-07 |
| GO:0016705 | oxidoreductase activity, acting on paired donors, with incorporation or reduction of molecular oxygen | 3.35E-08 | 8.25E-07 |
| GO:0005149 | interleukin-1 receptor binding | 4.38E-08 | 9.62E-07 |
| GO:0055085 | transmembrane transport | 4.39E-08 | 9.62E-07 |
| GO:0008509 | anion transmembrane transporter activity | 8.40E-08 | 1.74E-06 |
| GO:0005245 | voltage-gated calcium channel activity | 1.43E-07 | 2.61E-06 |
| GO:0005891 | voltage-gated calcium channel complex | 1.43E-07 | 2.61E-06 |
| GO:0016020 | membrane | 1.46E-07 | 2.61E-06 |
| GO:0070588 | calcium ion transmembrane transport | 3.01E-07 | 5.15E-06 |
| GO:0005391 | sodium:potassium-exchanging ATPase activity | 7.40E-07 | 1.17E-05 |
| GO:0030212 | hyaluronan metabolic process | 7.40E-07 | 1.17E-05 |
| GO:0006810 | transport | 2.57E-06 | 3.89E-05 |
| GO:0004623 | phospholipase A2 activity | 3.24E-06 | 4.40E-05 |
| GO:0007219 | Notch signaling pathway | 3.24E-06 | 4.40E-05 |
| GO:0050482 | arachidonic acid secretion | 3.24E-06 | 4.40E-05 |
| GO:0006814 | sodium ion transport | 6.64E-06 | 8.73E-05 |
| GO:0005576 | extracellular region | 7.95E-06 | 1.01E-04 |
| GO:0005216 | ion channel activity | 1.08E-05 | 1.27E-04 |
| GO:0006644 | phospholipid metabolic process | 1.09E-05 | 1.27E-04 |
| GO:0016702 | oxidoreductase activity, acting on single donors with incorporation of molecular oxygen, incorporation of two atoms of oxygen | 1.09E-05 | 1.27E-04 |
| GO:0006596 | polyamine biosynthetic process | 1.25E-05 | 1.40E-04 |
| GO:0016042 | lipid catabolic process | 1.84E-05 | 2.02E-04 |
| GO:0006952 | defense response | 2.23E-05 | 2.38E-04 |
| GO:0006811 | ion transport | 3.15E-05 | 3.26E-04 |
| GO:0020037 | heme binding | 3.64E-05 | 3.67E-04 |
| GO:0005579 | membrane attack complex | 5.94E-05 | 5.20E-04 |
| GO:0006406 | mRNA export from nucleus | 5.94E-05 | 5.20E-04 |
| GO:0006839 | mitochondrial transport | 5.94E-05 | 5.20E-04 |
| GO:0009922 | fatty acid elongase activity | 5.94E-05 | 5.20E-04 |
| GO:0019367 | fatty acid elongation, saturated fatty acid | 5.94E-05 | 5.20E-04 |
| GO:0042761 | very long-chain fatty acid biosynthetic process | 5.94E-05 | 5.20E-04 |
| GO:0004190 | aspartic-type endopeptidase activity | 9.14E-05 | 7.66E-04 |
| GO:0007156 | homophilic cell adhesion via plasma membrane adhesion molecules | 9.07E-05 | 7.66E-04 |
| GO:0004197 | cysteine-type endopeptidase activity | 1.38E-04 | 1.13E-03 |
| GO:0008289 | lipid binding | 1.54E-04 | 1.24E-03 |
| GO:0004176 | ATP-dependent peptidase activity | 1.70E-04 | 1.31E-03 |
| GO:0030154 | cell differentiation | 1.70E-04 | 1.31E-03 |
| GO:0001733 | galactosylceramide sulfotransferase activity | 2.10E-04 | 1.40E-03 |
| GO:0005219 | ryanodine-sensitive calcium-release channel activity | 2.10E-04 | 1.40E-03 |
| GO:0006874 | cellular calcium ion homeostasis | 2.10E-04 | 1.40E-03 |
| GO:0008191 | metalloendopeptidase inhibitor activity | 2.10E-04 | 1.40E-03 |
| GO:0008308 | voltage-gated anion channel activity | 2.10E-04 | 1.40E-03 |
| GO:0009247 | glycolipid biosynthetic process | 2.10E-04 | 1.40E-03 |
| GO:0030866 | cortical actin cytoskeleton organization | 2.10E-04 | 1.40E-03 |
| GO:0044070 | regulation of anion transport | 2.10E-04 | 1.40E-03 |
| GO:0016817 | hydrolase activity, acting on acid anhydrides | 2.52E-04 | 1.66E-03 |
| GO:0005886 | plasma membrane | 2.64E-04 | 1.71E-03 |
| GO:0007166 | cell surface receptor signaling pathway | 2.95E-04 | 1.87E-03 |
| GO:0005337 | nucleoside transmembrane transporter activity | 3.77E-04 | 2.25E-03 |
| GO:0015280 | ligand-gated sodium channel activity | 3.77E-04 | 2.25E-03 |
| GO:0031966 | mitochondrial membrane | 3.77E-04 | 2.25E-03 |
| GO:0051262 | protein tetramerization | 3.77E-04 | 2.25E-03 |
| GO:0071805 | potassium ion transmembrane transport | 4.37E-04 | 2.57E-03 |
| GO:0007155 | cell adhesion | 4.53E-04 | 2.62E-03 |
| GO:0004888 | transmembrane signaling receptor activity | 6.12E-04 | 3.49E-03 |
| GO:0004866 | endopeptidase inhibitor activity | 6.36E-04 | 3.58E-03 |
| GO:0004842 | ubiquitin-protein transferase activity | 7.32E-04 | 4.06E-03 |
| GO:0004683 | calmodulin-dependent protein kinase activity | 8.03E-04 | 4.39E-03 |
| GO:0005267 | potassium channel activity | 1.10E-03 | 5.91E-03 |
| GO:0005272 | sodium channel activity | 1.23E-03 | 6.48E-03 |
| GO:0006887 | exocytosis | 1.23E-03 | 6.48E-03 |
| GO:0008234 | cysteine-type peptidase activity | 1.57E-03 | 8.17E-03 |
| GO:0016810 | hydrolase activity, acting on carbon-nitrogen (but not peptide) bonds | 1.86E-03 | 9.39E-03 |
| GO:0031418 | L-ascorbic acid binding | 1.86E-03 | 9.39E-03 |
| GO:0008023 | transcription elongation factor complex | 1.92E-03 | 9.57E-03 |
| GO:0000145 | exocyst | 1.96E-03 | 9.65E-03 |
| GO:0030414 | peptidase inhibitor activity | 2.93E-03 | 1.43E-02 |
| GO:0004867 | serine-type endopeptidase inhibitor activity | 3.38E-03 | 1.53E-02 |
| GO:0006935 | chemotaxis | 3.38E-03 | 1.53E-02 |
| GO:0008009 | chemokine activity | 3.38E-03 | 1.53E-02 |
| GO:0008158 | hedgehog receptor activity | 3.54E-03 | 1.53E-02 |
| GO:0015276 | ligand-gated ion channel activity | 3.54E-03 | 1.53E-02 |
| GO:0031047 | gene silencing by RNA | 3.54E-03 | 1.53E-02 |
| GO:0046373 | L-arabinose metabolic process | 3.54E-03 | 1.53E-02 |
| GO:0046556 | alpha-L-arabinofuranosidase activity | 3.54E-03 | 1.53E-02 |
| GO:0050793 | regulation of developmental process | 3.54E-03 | 1.53E-02 |
| GO:0051028 | mRNA transport | 3.54E-03 | 1.53E-02 |
| GO:0006368 | transcription elongation from RNA polymerase II promoter | 3.67E-03 | 1.55E-02 |
| GO:0015631 | tubulin binding | 3.67E-03 | 1.55E-02 |
| GO:0005741 | mitochondrial outer membrane | 5.78E-03 | 2.42E-02 |
| GO:0042981 | regulation of apoptotic process | 6.06E-03 | 2.51E-02 |
| GO:0030695 | GTPase regulator activity | 6.13E-03 | 2.52E-02 |
| GO:0006508 | proteolysis | 6.31E-03 | 2.56E-02 |
| GO:0003779 | actin binding | 7.58E-03 | 3.05E-02 |
| GO:0004872 | receptor activity | 8.60E-03 | 3.41E-02 |
| GO:0007275 | multicellular organismal development | 8.65E-03 | 3.41E-02 |
| GO:0004013 | adenosylhomocysteinase activity | 1.02E-02 | 3.72E-02 |
| GO:0004332 | fructose-bisphosphate aldolase activity | 1.02E-02 | 3.72E-02 |
| GO:0004645 | phosphorylase activity | 1.02E-02 | 3.72E-02 |
| GO:0004887 | thyroid hormone receptor activity | 1.02E-02 | 3.72E-02 |
| GO:0008140 | cAMP response element binding protein binding | 1.02E-02 | 3.72E-02 |
| GO:0008184 | glycogen phosphorylase activity | 1.02E-02 | 3.72E-02 |
| GO:0019510 | S-adenosylhomocysteine catabolic process | 1.02E-02 | 3.72E-02 |
| GO:0032947 | protein complex scaffold | 1.02E-02 | 3.72E-02 |
| GO:0005634 | nucleus | 1.06E-02 | 3.78E-02 |
| GO:0008270 | zinc ion binding | 1.05E-02 | 3.78E-02 |
| GO:0004725 | protein tyrosine phosphatase activity | 1.09E-02 | 3.86E-02 |
| GO:0007165 | signal transduction | 1.17E-02 | 4.13E-02 |
| GO:0006816 | calcium ion transport | 1.22E-02 | 4.21E-02 |
| GO:0016567 | protein ubiquitination | 1.22E-02 | 4.21E-02 |
| GO:0006955 | immune response | 1.28E-02 | 4.37E-02 |
| GO:0015671 | oxygen transport | 1.35E-02 | 4.57E-02 |
| GO:0006334 | nucleosome assembly | 1.40E-02 | 4.69E-02 |
| GO:0016311 | dephosphorylation | 1.40E-02 | 4.69E-02 |
