# Supplementary material for: The First High-quality Reference Genome of Sika Deer Provides Insights into High-tannin Adaptation
Source: Genomics Proteomics Bioinformatics. 2022 Jun 16;21(1):203–15. doi: 10.1016/j.gpb.2022.05.008 (PMC10372904; doi:10.1016/j.gpb.2022.05.008)
Supplement: Supplementary Table S19 [file mmc36.docx]

**Table S19**  **Numbers of annotated *UGT* genes in 19 species**

|  | **UGT2B** | **UGT2C** | **UGTA3** | **UGT2A1** | **UGT1A** | **UGT8** | **UGT3A** | **Total** |
| --- | --- | --- | --- | --- | --- | --- | --- | --- |
| Human | 7 | 0 | 1 | 1 | 1 | 1 | 2 | 13 |
| Mouse | 7 | 0 | 1 | 1 | 1 | 1 | 2 | 13 |
| Single-humped camel | 3 | 2 | 0 | 1 | 1 | 1 | 1 | 9 |
| Double-humped camel | 1 | 1 | 0 | 1 | 1 | 1 | 1 | 6 |
| Pig | 4 | 1 | 1 | 1 | 1 | 0 | 1 | 9 |
| Minke whale | 1 | 1 | 1 | 0 | 1 | 1 | 1 | 6 |
| Okapi | 4 | 5 | 1 | 0 | 1 | 1 | 1 | 13 |
| Giraffe | 6 | 2 | 0 | 0 | 1 | 1 | 1 | 11 |
| Musk deer | 3 | 2 | 0 | 0 | 3 | 1 | 1 | 10 |
| Cattle | 4 | 3 | 0 | 1 | 1 | 1 | 2 | 12 |
| Yak | 6 | 5 | 1 | 0 | 1 | 1 | 1 | 15 |
| Goat | 7 | 4 | 0 | 1 | 1 | 1 | 2 | 16 |
| Sheep | 5 | 3 | 1 | 0 | 1 | 1 | 2 | 13 |
| Roe deer | 4 | 3 | 0 | 0 | 1 | 1 | 1 | 10 |
| Reindeer | 8 | 3 | 1 | 0 | 1 | 0 | 1 | 14 |
| White-tailed deer | 6 | 4 | 0 | 1 | 5 | 1 | 2 | 19 |
| Milu | 6 | 4 | 1 | 0 | 2 | 1 | 2 | 16 |
| Red deer | 13 | 6 | 3 | 0 | 1 | 0 | 2 | 25 |
| Sika deer | 15 | 5 | 1 | 2 | 2 | 0 | 2 | 27 |
