# Supplementary material for: The First High-quality Reference Genome of Sika Deer Provides Insights into High-tannin Adaptation
Source: Genomics Proteomics Bioinformatics. 2022 Jun 16;21(1):203–15. doi: 10.1016/j.gpb.2022.05.008 (PMC10372904; doi:10.1016/j.gpb.2022.05.008)
Supplement: Supplementary Table S20 [file mmc37.docx]

**Table S20**  **Design of the feeding experiment**

|  | **0 MOL**  **(y0 group)** | **50% MOL**  **(y50 group)** | **100% MOL**  **(y100 group)** | **100% MOL**  **(m100 group)** |
| --- | --- | --- | --- | --- |
| Sika deer | 15 tissues*3 | 15 tissues*3 | 15 tissues*3 | 15 tissues*3 |
|  | 0 GA (n group) | | 10% GA (y group) | |
| Cattle | 8 tissues*3^1^ | | 8 tissues*3 | |

*Note*: 9 young sika were divided into three groups in different diet: didn’t feed MOL, fed 50% MOL, 100% MOL, and 3 adult sika deer fed 100% MOL as the fourth group. For comparison 6 cattle were divided into two groups in different diet: didn’t feed gallotannic acid (GA) and fed 10% GA. Cattle usually doesn’t eat MOL because of acerbity taste derived from high level tanin. So GA was used instead of MOL. Twelve sika deer were divided into four groups. Six cattle were divided into two groups. ^1^ Two samples were not sequenced due to the pool quality. GA, gallotannic acid; MOL, Mongolian oak leaves.
